# Fetal Fraction Signatures: A Quality Control Tool to Detect Potentially Confounding Situations in NonInvasive Prenatal Diagnosis of Monogenic Conditions

**DOI:** 10.1111/cge.70121

**Published:** 2025-12-09

**Authors:** Jean‐Louis Blouin, Claudine Rieubland, Thierry Nouspikel

**Affiliations:** ^1^ Genetic Medicine, Diagnostic Department Geneva University Hospitals Geneva Switzerland; ^2^ Department of Genetic Medicine and Development, Faculty of Medicine University of Geneva Geneva Switzerland; ^3^ Department of Medical Genetics, Central Institute of the Hospitals Hospital of the Valais Sion Switzerland

**Keywords:** circulating cell‐free DNA, fetal DNA, monogenic disease, noninvasive prenatal diagnosis

## Abstract

Noninvasive prenatal diagnosis relies on the analysis of the small amount of cell‐free fetal DNA circulating in maternal plasma. Widely used to screen for chromosomal anomalies, the technique can also be applied to Mendelian diseases (NIPD‐M). However, asserting the presence of a maternal variant in the fetus is challenging and requires the determination of the maternal haplotypes transmitted to the fetus. This is achieved via relative haplotype dosage (RHDO), a method that relies on the analysis of the allelic balance of multiple single‐nucleotide polymorphisms around the pathogenic variant. An inherent risk of this method is that twin pregnancies and chromosomal anomalies like trisomy, monosomy or uniparental disomy unavoidably alter allelic balance, potentially leading to incorrect diagnosis. Here, we introduce an analytical method, fetal fraction signatures, that can detect such confounding situations in NIPD‐M data, without the need for additional wet‐lab work. We show that our method can reliably detect anomalies down to a low level of mosaicism and a low fetal fraction, thereby providing an essential quality‐control tool for clinical testing by NIPD‐M.

## Graphical abstract

Noninvasive prenatal diagnosis for monogenic diseases (NIPD‐M) to detect inherited mutations is performed in parallel with fetal fraction signatures, to detect various anomalies that may compromise diagnosis. An extra regression analysis may be necessary to resolve mosaic situations.

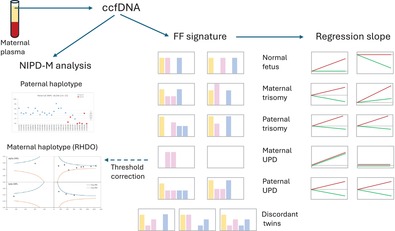

## 1 Introduction

Noninvasive prenatal tests (NIPT) have become increasingly popular in the past decade and are now part of routine pregnancy management in many countries, as they do not entail a risk of miscarriage [1]. These tests are based on the analysis of circulating cell‐free DNA (ccfDNA) in maternal plasma and rely on the presence of a minor fraction of fetal ccfDNA of placental origin, within maternal ccfDNA [2]. The technique was initially used to screen for chromosomal aneuploidies (NIPT‐A), but in the past few years several clinical diagnostic laboratories began to implement it as a diagnostic assay for monogenic diseases (NIPD‐M), mostly to assess the presence of known parental mutations in the fetus [3, 4, 5].

While the detection of a paternal mutation in maternal plasma is relatively straightforward, determining the presence or absence of a maternal or biparental mutation in fetal ccfDNA is unavoidably complicated by the overwhelming amount of maternal ccfDNA carrying the same variant. An elegant solution, relative haplotype dosage (RHDO), has become the standard method to indirectly assess the presence of a mutation by determining which parental haplotype was transmitted to the fetus [6]. In practice, RHDO is achieved by genotyping a small number of frequent single‐nucleotide polymorphisms (SNPs) on either side of the pathogenic variant.

Depending on their disposition in the parents, SNPs on the autosomes can be classified into five types, serving different purposes [6]. Type‐1 SNPs are those for which the parents are both homozygous, but for different alleles. The fetus is thus obligatorily heterozygous and the frequency of the paternal allele in ccfDNA serves to determine fetal fraction (FF), that is, the fraction of ccfDNA of fetal origin. Type‐2 SNPs, for which both parents are homozygous for the same allele, can be used to determine background noise, although any invariant position may serve as well for this purpose. Type‐3 SNPs, for which the father is heterozygous and the mother homozygous, allow us to determine which paternal haplotype was transmitted to the fetus, a requirement when both parents carry the same mutation. These SNPs are subdivided into alpha and beta SNPs, depending on which of the two paternal haplotypes carries an allele that is absent in the mother. Similarly, Type‐4 SNPs, for which the mother is heterozygous and the father homozygous, are divided into alpha and beta subtypes, depending on whether the homozygous allele in the father corresponds to maternal haplotype M1 (alpha) or M2 (beta). Type‐4 SNPs form the basis of RHDO and are crucial to determine which maternal haplotype has been transmitted to the fetus and is therefore overrepresented in maternal plasma. Finally, we have shown that Type‐5 SNPs, for which both parents are heterozygous, can enable NIPD‐M in consanguineous couples, which is otherwise challenging as the necessary Type‐3 and Type‐4 SNPs do not exist in regions of identity‐by‐descent frequently observed in such couples [7].

The reliance of the technique on allelic frequencies or (for Type‐4 and Type‐5 SNPs) on the allelic balance makes it highly sensitive to alterations in dosage. Monosomy or trisomy of the target chromosome, homogeneous, or in mosaic, unavoidably alters allelic balance, potentially making diagnosis impossible, or worse leading to an incorrect conclusion. This is also the case for uniparental disomies, of paternal or maternal origin, and for dizygotic twin pregnancies. Figure S1 illustrates the case of an unrecognized vanishing twin, as the likely cause of a very convincing but incorrect result.

The ability to detect such situations is therefore critically important to ensure the reliability of NIPD‐M diagnostic results. While this could be achieved by pairing the test with a standard NIPT‐A test, it would be advantageous if a single test could provide both types of information. Here, we introduce “fetal‐fraction signatures,” a technique which, without any additional wet‐lab requirements, allows for reliable detections of most anomalies that could potentially interfere with NIPD‐M results.

## 2 Methods

To simulate potentially confounding situations, such as monosomy, trisomy, or uniparental disomy, we mixed DNA from selected male samples and assayed the resulting samples with a panel of SNPs located on the X chromosome, in 1 Mbp‐wide regions surrounding the F8, FMR1 and TAF1 loci and part of the DMD locus. Although such samples are di‐, tetra‐, or hexaploid, they contain the required number of X chromosomes, and the rest of the genome is invisible to the analysis. We then simulated prenatal samples by mixing a “fetal” sample with a corresponding “maternal” sample. Finally, twin pregnancies and mosaic situations were simulated by mixing two “fetal” samples in various proportions within “maternal” DNA (Figure S2).

Libraries were constructed as previously described [8] from 50 ng DNA, with a system featuring molecular barcodes and suitable for both ccfDNA and genomic DNA (QIAseq, Qiagen), targeting 389 SNPs located on the X chromosome and four loci on the Y chromosome. Libraries were sequenced on a NextSeq500 sequencer (Illumina) as 2 × 75 nucleotides, with an average sequencing depth of 1595× and an average molecular depth of 640×. Results were analyzed with the smCounter2 pipeline [9], using Fgbio consensus option, genotype was determined with BamReadcount and further calculations were performed in Excel (see supplementary methods for details).

**FIGURE 1:**
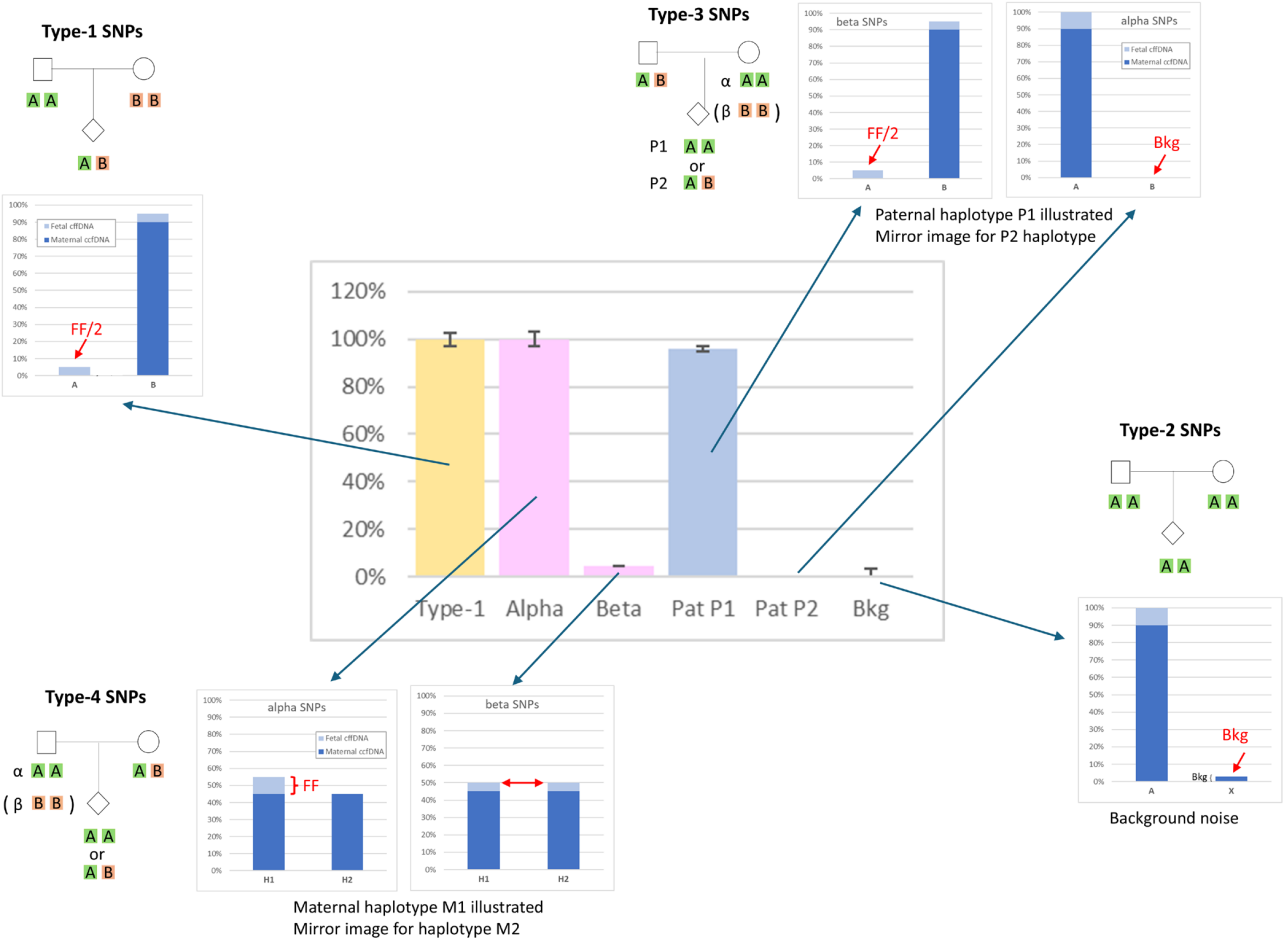
Principle of fetal fraction signatures. Fetal fraction is calculated with different SNP types: (i) Type‐1 SNPs, for which both parents are homozygous for different alleles. FF (yellow bar) is twice the frequency of the paternal allele. Error bars are the average deviation between the frequency of individual SNPs. (ii) Type‐3 SNPs, for which the father is heterozygous and the mother homozygous, should yield the same FF value as type‐1 SNPs for paternal alleles located on the haplotype transmitted to the fetus (P1 in this example). The other SNPs should yield a null value, or inferior to background noise. FF (blue bars) is twice the frequency of the paternal allele. (iii) Type‐2 SNPs, for which both parents are homozygous for the same allele, can be used to determine background noise (average frequency of the 2nd most frequent alleles). (iv) Type‐4 SNPs, for which the mother is heterozygous and the father homozygous, should yield the same FF value for one SNP subtype (alpha or beta, depending on which maternal haplotype was transmitted to the fetus). The other subtype should yield a null value. FF (pink bars) is the difference between maternal alleles belonging to haplotype M1 and alleles belonging to haplotype M2. In this example, the fetus inherited maternal haplotype M1, which is thus overrepresented in alpha SNPs only.

To detect the presence of an anomaly, we calculated FF using Type 1, 3, and 4 SNPs, and compared the values obtained with these different SNP types (Figure 1). For Type‐1 SNPs, FF is twice the frequency of the paternal allele [6]. This is also true for Type‐3 SNPs, but only if the paternal‐specific allele is located on the haplotype received by the fetus. For the other Type‐3 subtype, allelic frequency is expected to be null or at least to fall within sequencing noise, as appraised with Type‐2 SNPs. For Type‐4 SNPs, the expected allelic balance is 50% if the fetus inherited different alleles from each parent, whereas an allele will be overrepresented when both parents transmitted it. The difference in allelic balance between haplotypes is thus equal to FF for one Type‐4 subtype and null for the other. In a normal pregnancy, identical values for FF are thus expected with Type‐1 SNPs, one subtype of Type‐3 SNPs, and one subtype of Type‐4 SNPs, whereas values calculated with the other subtypes of Type‐3 and Type‐4 SNPs should fall within background noise.

To further resolve small differences in allelic balance for Type‐4 SNPs, we reduced the effective FF by diluting in various proportions sequencing data from a prenatal sample with sequencing data from the corresponding maternal sample. The FF values calculated with Type‐4 alpha (positive) and Type‐4 beta SNPs (negative) were then plotted against those calculated with Type‐1 SNPs and linear regression coefficients were determined. In a normal situation, one subtype of Type‐4 SNPs should mirror Type‐1 SNPs, resulting in a regression slope of ±1, whereas the other subtype, oscillating within background noise, should yield a regression slope of 0.

## 3 Results

### 3.1 Specific FF Signatures for the Various Anomalies

**FIGURE 2:**
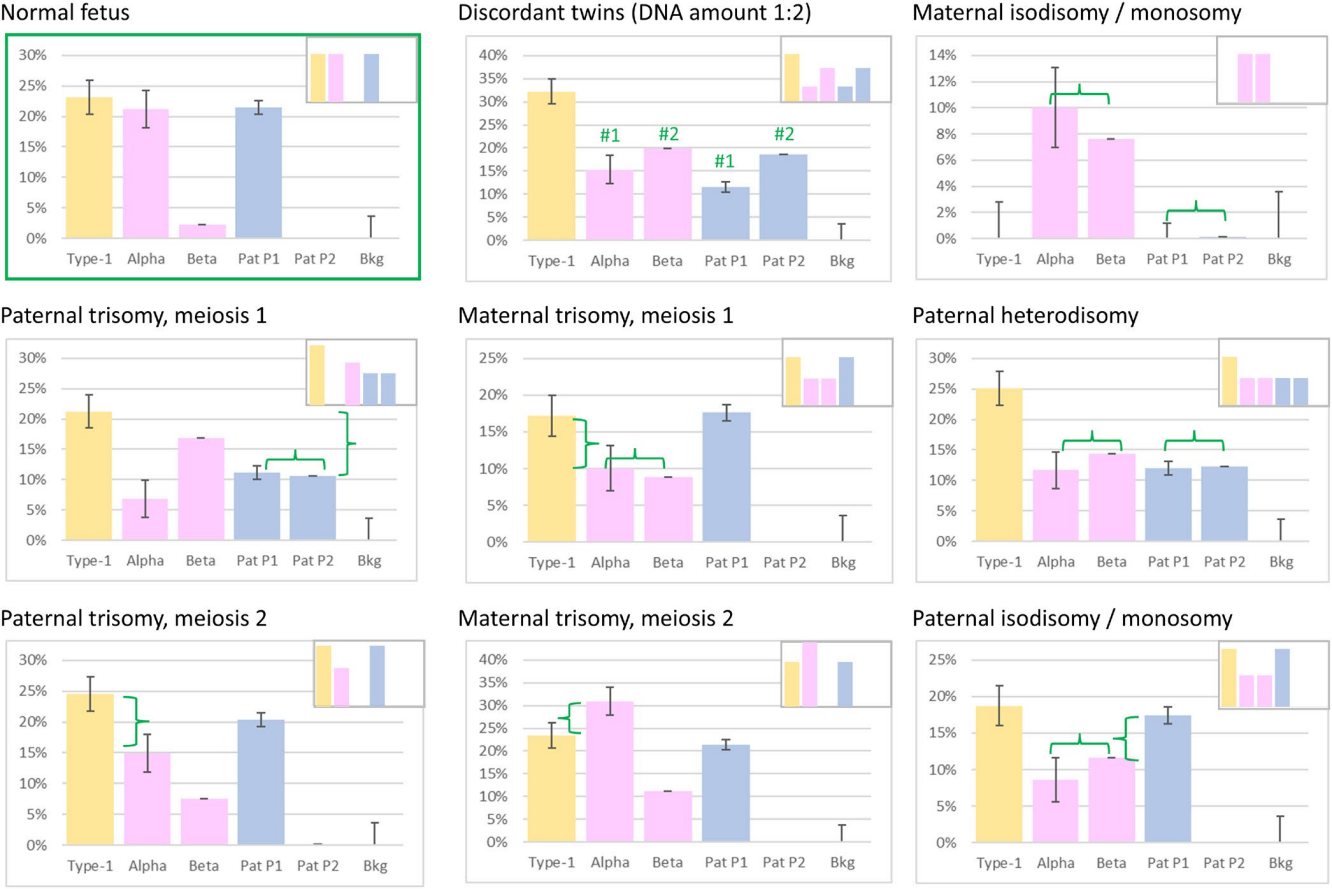
Specific fetal‐fraction signatures for the various anomalies. Composite samples simulating various abnormal prenatal situations were assayed with a panel of SNPs located on the X chromosome. In a normal situation (top left panel), identical FFs are measured with Type‐1 SNPs, one subtype of Type‐3 SNPs (paternal haplotype P1 or P2) and one subtype of Type‐4 SNPs (alpha or beta), the other two subtypes being inferior to background noise (Bkg, appraised with Type‐2 SNPs). This pattern was altered in a specific manner for each anomaly, inset cartoons depict the expected pattern. In each case, the key diagnostic features are denoted in green. Expected patterns are identical for monosomy and isodisomy, the samples tested here corresponded to monosomy. For the twin pregnancy sample, the haplotypes of each twin, contributing respectively 33% and 67% of fetal DNA, are labeled #1 and #2. Y‐axis: Calculated FF, error bars: Average deviation between individual SNPs.

We simulated various prenatal situations by mixing DNA from several male samples and assaying only loci located on the X chromosome. FF was calculated with Type‐1, Type‐3, and Type‐4 SNPs and sequencing noise was determined from Type‐2 SNPs. In a normal situation, nearly identical FF values were obtained from Type‐1 SNPs, one subtype of Type‐3 SNPs and one subtype of Type‐4 SNPs. The other Type‐3 and Type‐4 subtypes yielded values similar to background noise. As shown in Figures 2 and S3, this signature pattern was altered in specific manners by the various abnormal situations we considered.

Trisomies caused by nondisjunction in meiosis one result in the presence of two distinct chromosomes from the parent of origin. This situation was easily detectable for maternal (via Type‐4 SNPs) and paternal (via Type‐3 SNPs) trisomies, since both the alpha and beta subtypes yielded similar FF values, approximately half that of Type‐1 SNPs. Nondisjunction in meiosis two yields a trisomy with two identical chromosomes from the parent of origin. For maternal trisomies, this resulted in an FF value calculated from Type‐4 SNPs significantly higher than that calculated with Type‐1 or Type‐3 SNPs. Paternal trisomies affect both Type‐1 and Type‐3 SNPs, and the FF calculated with these SNPs thus appeared higher than that of Type‐4 SNPs. Verification of the “true” FF with SNPs located on other chromosomes would confirm that the correct value is that obtained with Type‐4 SNPs.

Twin pregnancies may or may not be detected, depending on concordance between twins. If each twin inherited a different paternal haplotype, both subtypes of Type‐3 SNPs are detectable, in proportions reflecting the relative contribution of each twin to the total amount of ccfDNA. The same pattern is detectable with Type‐4 SNPs when the twins inherited different maternal haplotypes. Figure 2 illustrates a situation where twins were divergent in both the maternal and paternal alleles, with one twin contributing twice as much ccfDNA as the other. As expected, both paternal haplotypes were detected with a 2:1 ratio, and so were both maternal haplotypes.

When twins are fully concordant, that is, inherited the same paternal and maternal haplotype at the locus of interest, the expected FF signature is undistinguishable from that of a normal singleton pregnancy. Conceivably, the presence of twins could be detected by testing SNPs located on other chromosomes, provided the twins are dizygotic. This is generally not necessary, however: since both twins have the same genotype for the gene of interest, RHDO results apply to both. The only potential problem would be a wide discrepancy in the amount of ccfDNA released by each twin, with the minor genotype being masked by the most abundant one. This situation is akin to mosaicism, which will be discussed below.

Uniparental disomies and monosomies yield typical patterns in which the contribution from one parent is absent. It is generally not possible to distinguish an isodisomy from a monosomy, unless FF is ascertained with SNPs located on a different chromosome: the apparent FF is expected to be half the real FF in the case of monosomy but should match it in the case of isodisomy. In paternal heterodisomy, the four subtypes of Type‐3 and Type‐4 SNPs yielded the same FF value, half that of Type‐1 SNPs. This was expected because, for Type‐4 SNPs, two identical (homozygous) paternal alleles are transmitted to the fetus, and both count toward haplotype M1 for alpha SNPs and haplotype M2 for beta SNPs (see File S1 for illustration and mosaicism calculations). In maternal heterodisomy (Figure S3), fetal and maternal genotypes are identical, and such samples cannot be distinguished from a purely maternal sample, such as that of a nonpregnant patient or a very early pregnancy with FF values inferior to the limit of detection. Again, this could be resolved by testing Type‐1 SNPs located on other chromosomes, to ascertain the presence of fetal DNA and determine the actual FF.

### 3.2 Effect of Mosaicism and Low Fetal Fraction

Since monosomies and most trisomies are not viable, one can expect that such situations shall generally be observed in mosaic, possibly confined to the placenta. Depending on the amount of mosaicism, mosaic aneuploidies may retain the ability to perturb RHDO results, and it is unfortunately likely that they shall be more difficult to detect than in the homogeneous state, particularly when FF is low.

To determine the effect of mosaicism and low FF on detection sensitivity, we generated composite samples by mixing an abnormal sample and a matching normal sample in various proportions, within a maternal sample. To reduce FF, we then diluted sequencing data obtained from this composite sample with data from the maternal sample.

**FIGURE 3:**
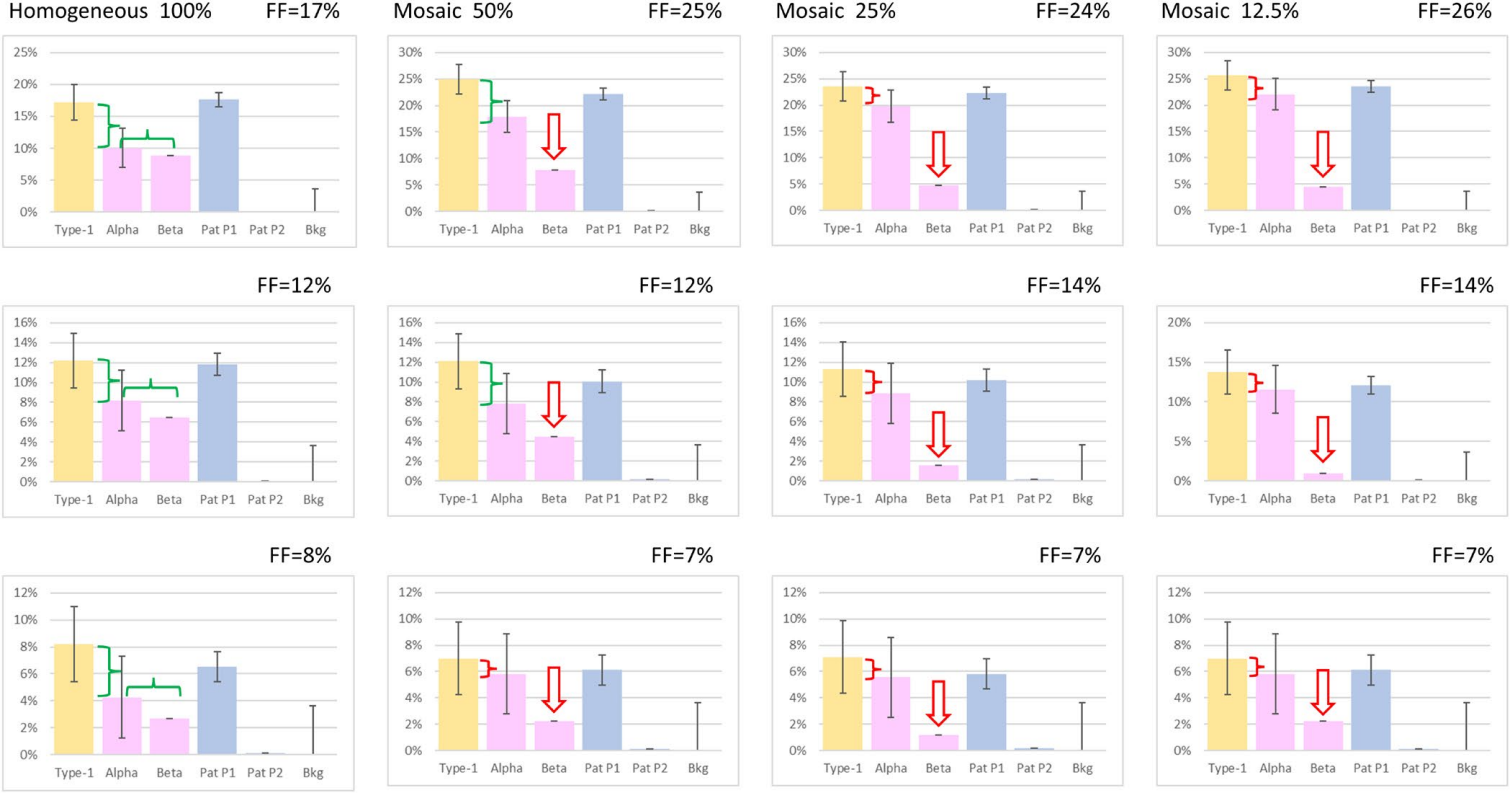
Effect of mosaicism and low FF. Composite samples were assembled is various proportions to simulate a maternal trisomy resulting from nondisjunction in meiosis 1. In columns, from left to right: Homogeneous trisomy, 50% mosaic, 25% mosaic and 12.5% mosaic. To reduce FF, sequencing data from each sample were mixed with sequencing data from the maternal sample, in various proportions. The key signature of a pure meiosis 1 trisomy is that FF calculated from alpha and beta SNPs yield identical values, approximately half of that calculated with Type‐1 or Type‐3 SNPs (upper left panel). This signature became increasingly difficult to recognize (denoted in red) as the amount of abnormal DNA was decreased, and when noise increased due to lower FF.

As expected, the typical diagnostic pattern of an anomaly became more difficult to recognize in mosaic situations (Figures S4–S19). Lowering FF did not alter the pattern but made it more difficult to distinguish from the normal pattern, as it decreased the signal‐to‐noise ratio. This effect was less of a problem for Type‐1 and Type‐3 SNPs, since it is relatively easy to detect low amounts of an allele that should not be present, at least if background noise is reasonably low. In this respect, it is important to select a library system featuring a low background, for instance by using molecular barcodes. By contrast, for Type‐4 SNPs, the detected event is a divergence from the 50:50 balance, which is much more sensitive to stochastic noise. Figure 3 demonstrates the effect of mosaicism and low FF on the detection of a maternal meiosis 1 trisomy, the most difficult situation to detect. Detection already became challenging with a 50:50 ratio of abnormal to normal DNA and was virtually impossible with 12.5% abnormal DNA. Low FF further compounded the problem by increasing the inter‐SNP coefficient of variation (error bars), thereby blurring the difference between normal and abnormal signals.

To overcome this problem, we elected to plot the FF measurements obtained with Type‐4 SNPs, alpha and beta separately, versus those obtained with Type‐1 SNPs and to calculate linear regression slopes for each subtype. In a normal sample, the slope should be +1 for alpha SNPs and 0 for beta SNPs if the fetus inherited maternal haplotype M1, whereas maternal haplotype M2 would result in a slope of 0 for alpha SNPs and—1 for beta SNPs. Most abnormal situations are expected to alter slope values for one or both subtypes, and the small stochastic variations hampering diagnosis should be smoothened by the regression process. Thus, the regression slopes should provide a more reliable indicator than the comparison of FFs calculated from Type‐1 and Type‐4 SNPs at a single point.

**FIGURE 4:**
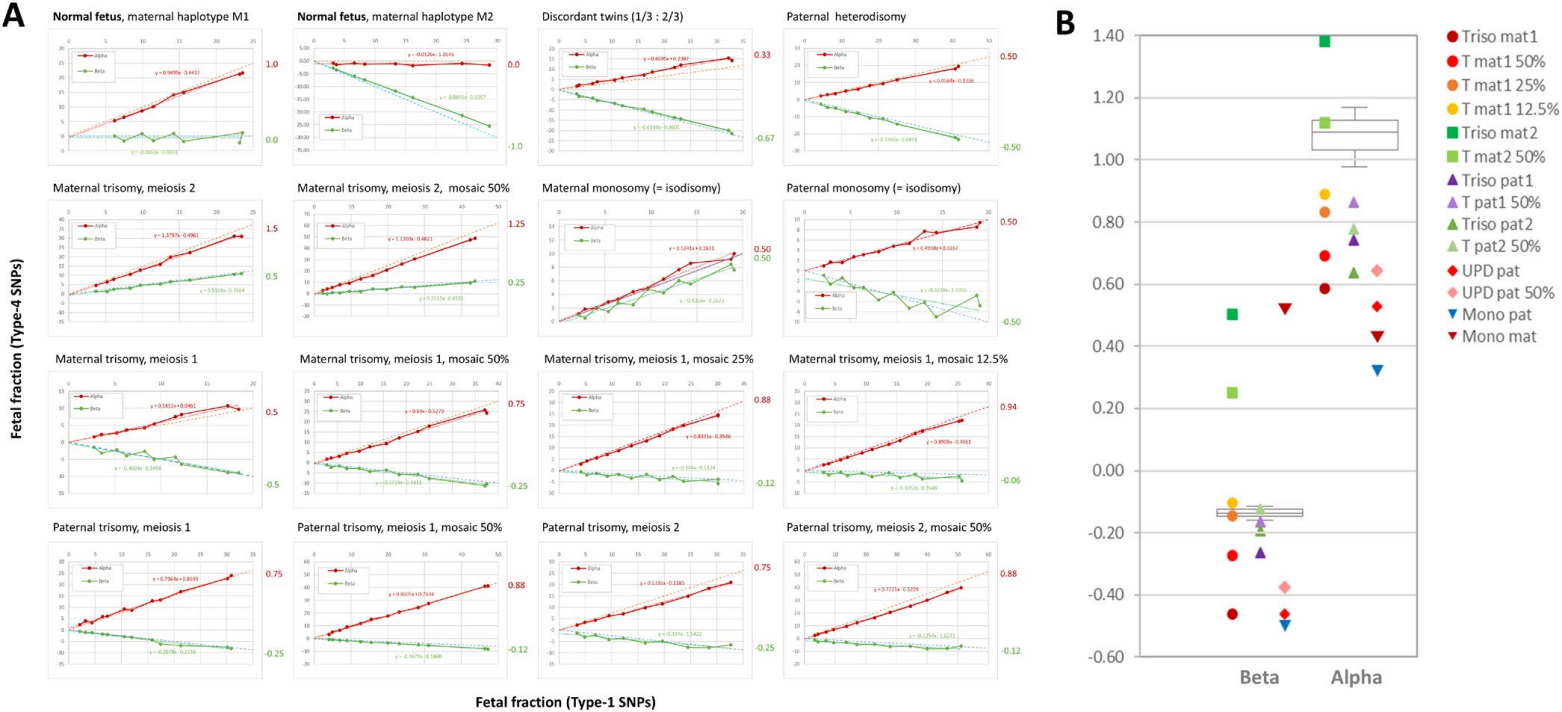
Refining the analysis of Type‐4 SNPs. (A) Data from composite samples corresponding to various anomalies were diluted with data from the corresponding maternal sample, to artificially reduce FF. The two FF values calculated with Type‐4 alpha (red) and beta (green) SNPs were plotted against the value calculated with Type‐1 SNPs, and linear regression slopes were calculated. For normal samples (first two graphs) the slope is ±1 for one SNP subtype and 0 for the other. All anomalies yielded abnormal slope values, indicated in the equations and in Table ST1 (expected values are displayed on the right of each graph). (B) Summary of regression slopes for alpha and beta SNPs for all anomalies. Boxes represent median and 1st/3rd quartiles for three genuine prenatal samples from normal pregnancies, whiskers are value range.

Indeed, Figure 4 and Table ST1 demonstrate that anomalies affecting maternal haplotypes were detectable down to a low (1 in 8) ratio of abnormal to normal mosaicism. While it occasionally happened that the slope for one subtype of Type‐4 SNPs fell within the normal range, the slope for the other subtype invariably allowed a solid conclusion (Figure 4B). Numeric anomalies affecting paternal haplotypes also altered the regression slopes of Type‐4 SNPs, but it was not always possible to distinguish an aneuploidy from another (e.g., both types of paternal trisomies are expected to yield identical regression slopes). In practice, this is not a problem since the distinction can easily be made from the analysis of Type‐3 SNPs, for which a regression strategy is not necessary.

## 4 Discussion

Here we introduce a technique we dubbed “fetal‐fraction signatures”, which we show can detect virtually all situations that may compromise the validity of NIPD‐M results: trisomies, monosomies, and uniparental disomies of the chromosome of interest, as well as dizygotic twin pregnancies. In a few cases when two abnormal situations cannot be distinguished via their signatures (e.g., monosomy and uniparental isodisomy), they nevertheless differ from the normal situation and a definitive determination of the type of anomaly can always be obtained by measuring FF with SNPs located on other chromosomes (implying however that such SNPs were included in the library design).

This analysis can also be applied to loci on the X chromosome, provided the library includes a few loci on chromosome Y to determine fetal gender and calculate FF in male fetuses. For a female fetus, FF signatures are similar to those of autosomal loci (Figure S20). For a male fetus, the expected signature pattern corresponds to that of a maternal monosomy and the presence of a paternal haplotype would indicate a 47,XXY genotype.

Our technique is robust and sensitive for most anomalies and allows definitive diagnosis even at low FF and in mosaic situations. The greatest challenge is aneuploidies of maternal origin, since their detection is based on Type‐4 SNPs, for which both alleles are necessarily present in maternal ccfDNA. Trisomies due to nondisjunction in meiosis two result in a double amount of the same haplotype, making haplotype determination by RHDO easier. By contrast, nondisjunction in meiosis one results in the presence of two different maternal haplotypes in fetal ccfDNA, implying a significant probability of RHDO failure or even of incorrect results. We show that bioinformatic reduction of FF and analysis of the resulting regression slopes overcomes this problem and allows reliable detection of maternal trisomies down to 12.5% mosaicism. As the generation of FF signatures is based on the same data as the RHDO test, we reasoned that any anomaly in a mosaic ratio too low to be detected is unlikely to significantly perturb RHDO results.

An obvious alternative to our approach would be to perform a traditional NIPT‐A test in parallel with every NIPD‐M test. This solution presents several drawbacks, though. Firstly, commercially available NIPT‐A tests based on SNPs often do not cover all chromosomes and may thus be useless for genes located on a chromosome not included in the panel. Those relying on very low pass genome sequencing can potentially target the entire genome (although the manufacturer may only have validated the dosage of a few selected chromosomes) but are typically unable to detect uniparental disomies. Second, no matter the NIPT‐A technique used, performing two tests in parallel implies higher costs and larger amounts of ccfDNA. The amount of input ccfDNA is key to achieving a high sensitivity in NIPD‐M, but ccfDNA levels widely vary between patients and ccfDNA yield may become a limiting factor, especially early in pregnancy, when FF is low. As our method relies on a supplementary analysis of existing data, it does not require extra ccfDNA, nor does it imply additional costs.

The presence of an aneuploidy of the target chromosome does not necessarily prevent NIPD‐M analysis, provided suitable corrections are introduced in the equations underlying the RHDO algorithm. We provide these equations in the supplementary methods and Tables ST2–ST5, although we did not attempt to validate them clinically. We reasoned that any aneuploidy detected in the placenta, and possibly confirmed by subsequent NIPT‐A, will likely prompt amniocentesis to assess the status of the fetus. The invasive sample can thus be used to assert the presence or absence of the familial mutation. In a mosaic situation, however, advanced determination of the mutational status of the normal cells may assist in the decision to proceed or not with amniocentesis.

A limitation of our work is that we were only able to test our method on a single abnormal sample, a probable vanishing twin pregnancy for which the presence of two paternal haplotypes was detected on two distinct chromosomes (Figure S1). Systematic analysis of prior cases revealed possible mosaic trisomies in two other samples, but we were not able to verify these suspicions in retrospect (Figure S21). To validate detection of other abnormal situations, we resorted to assembling composite samples with DNA from male patients and testing SNPs located on the X chromosome. However, as far as the technique is concerned, there is no difference between the X chromosome and any autosome. Our results can thus safely be extrapolated to other chromosomes, as demonstrated in the above case that implicated the *GCK* gene, on chromosome 7.

A future challenge will be to define cutoff criteria to allow automated detection of potentially confounding situations. For this purpose, we are tentatively using the standard deviation of the allelic frequencies across all SNPs of a given subtype and raise an alert when the mean ±1 SD of Type‐3 or Type‐4 SNPs does not fall within 1 SD of the mean of the control SNPs (Figure S21). Yet, due to our still limited experience with this metric, we feel that careful assessment of FF signatures and, if necessary, of regression slopes by an experienced scientist currently remains necessary.

In conclusion, we introduce a simple and reliable method, fetal‐fraction signatures, to detect anomalies that may compromise NIPD‐M results. As our technique uses the same sample and the same data as NIPD‐M, it does not imply additional wet‐lab work, nor extra costs. Considering the potentially devastating consequences of an incorrect prenatal diagnosis, a simple quality‐control step reliably flagging the most common biological sources of error is undeniably a valuable and welcome addition to this powerful technique.

## Conflicts of Interest

The authors declare no conflicts of interest.

## Supporting information


**Data S1:** cge70121‐sup‐0001‐SupFile1.xlsx.

**Data S2:** cge70121‐sup‐0002‐SupFile2.docx.

**Data S3:** cge70121‐sup‐0003‐Figures.pptx.

## Data Availability

The data that support the findings of this study are available from the corresponding author upon reasonable request.
