# Supplementary material for: Fetal Fraction Signatures: A Quality Control Tool to Detect Potentially Confounding Situations in NonInvasive Prenatal Diagnosis of Monogenic Conditions
Source: Clin Genet. 2025 Dec 9;109(5):869–75. doi: 10.1111/cge.70121 (PMC13066760; doi:10.1111/cge.70121)
Supplement: Supplementary file 2 — Data S2: cge70121‐sup‐0002‐SupFile2.docx. [file CGE-109-869-s003.docx]

# Detailed methods

Samples and DNA extraction

All patients gave consent for their samples to be used for the purpose of method development and analytical quality improvement.

Genomic DNA was extracted from venous blood using a QIASymphony robotic extractor and reagents (Qiagen). Circulating cell-free DNA was extracted from maternal venous blood collected into Streck BCT tubes (Streck) generally between 12 weeks and 16 weeks since last monthly period. Plasma was prepared by centrifugation for 10 min at 1'600 x g, 4°C, collected and centrifuged 10 min at 16'000 x g, 4°C. DNA was extracted from plasma with the QIAamp MinElute ccfDNA kit (Qiagen) according to manufacturer's instructions and quantified with a Qubit fluorimeter (Molecular Probes).

Composite samples

To simulate abnormal situations like trisomy, monosomy or uniparental disomy of the X chromosome, genomic DNA from male patients was mixed in equal amounts (e.g. 3 samples for a trisomy). Allelic balance was verified by sequencing (fig. S3), then prenatal samples were simulated by mixing the composite sample for a given anomaly with a genetically matched maternal sample (also a composite of two male samples), aiming for a fetal fraction of 20%, taking ploidy into account. To simulate mosaic situations, two composite samples, one with an anomaly and the matching normal sample, were mixed in various proportions with the corresponding maternal sample. The same strategy was used to simulate twin pregnancies. See supplementary figure S2 for illustration.

Library construction

QIASeq custom targeted libraries were built from 50 ng genomic DNA, using the manufacturer's protocol for genomic DNA and from 10 to 50 ng ccfDNA, using the circulating DNA protocol. Libraries were quantified by fluorimetry, and size profiles were verified with a Fragment Analyzer (Agilent).

Sequencing

Samples were pooled and sequenced as 2 x 75 nucleotides with a NextSeq500 sequencer (Illumina). In average, 3 million reads were obtained per sample (range 1.9 to 5.1 million). Depending on samples, average read depth was 964x to 2667x and average molecular depth was 410x to 1036x.

Analysis

Qiagen smCounter2 pipeline (Xu et al. Bioinformatics, 2019) was used to align and filter reads, deduplicate barcodes, and build consensus reads using Fgbio subroutines (https://github.com/fulcrumgenomics/fgbio). Allele counts were extracted from the resulting BAM files with bam-readcount (<https://github.com/genome/bam-readcount>).

Haplotypes were reconstructed from the genotype of the parents and that of a prior conceptus of this couple. When no such sample was available, haplotypes were only determined in the person carrying the mutation, by genotyping both grandparents.

RHDO was performed in Excel, as described by Lo *et al*. (*Sci Translat Med* 2010). Alpha and beta SNPs located upstream and downstream of the mutation were analyzed separately to detect the unlikely occurrence of meiotic recombination within the region of interest. Once this possibility was excluded, a global likelihood ratio was calculated for all alpha SNPs or for all beta SNPs, depending on which yielded the highest statistical significance.

FF signatures were obtained from the allelic frequencies of type-1, type-3 alpha and beta and type-4 alpha and beta SNPs. Allelic frequencies were calculated by cumulating molecular counts for all SNPs of a given (sub)type. Average deviation was obtained from the allelic frequencies of individual SNPs of the same subtype. Background noise was taken from type-2 SNPs. For type-1 and type-3 SNPs, FF was calculated as twice the frequency of the unique paternal allele (absent in the mother). For type-4 SNPs, FF was calculated as the absolute value of the difference between the frequency of unique maternal alleles (absent in the father) belonging to maternal haplotype M1 and those belonging to maternal haplotype M2.

Reducing fetal fraction

To achieve molecular downsampling, a custom filter was introduced in the smCounter2 pipeline to randomly reject a given fraction of molecules. This pipeline begins by gathering reads that share the same barcode and map at the same approximate location (indicating that they originate from the same DNA molecule), then several built-in quality checks can reject a read or a barcode. An extra filter was added at this step, to discard an arbitrarily chosen fraction of barcodes, despite good quality scores.

In script umi_mark.py, two identical lines with the following statement:

handleOneMolecule(alignments, fileout1, fileout2)

were modified as:

if counter%downsampling == 0:

handleOneMolecule(alignments, fileout1, fileout2)

counter += 1

Where *counter* is initialized upon entry and incremented for each new barcode, and *downsampling* was set to 1 to disable filtering or to a larger N value to accept only 1 read out of N.

BAM files from plasma sequencing data and maternal sequencing data, possibly downsampled, were then merged in various proportion with samtools merge. The lists of primers detected by the pipeline was merged with a “cat” command and the smCounter2 pipeline was resumed at the primer clip stage.

FF calculated with type-4 SNPs was plotted against that calculated with type-1 SNPs. Since no absolute value was applied here, alpha SNPs yielded positive values and beta SNPs negative values. Linear regression was performed for each subtype and the slope of the regression line was determined.

Modified RHDO calculations

Aneuploidy does not necessarily make RHDO impossible, but detection thresholds must be adjusted to account for changes in the contribution of parental haplotypes to fetal ccfDNA.

Lo and coworkers defined q1 as the ratio of maternal haplotype 1, M1/(M1+M2), when the fetus inherited maternal haplotype M1, whereas q0 is the same ratio when the fetus inherited maternal haplotype M2. Both q1 and q0 differ for type-4 alpha and type-4 beta. In a normal pregnancy, q1 is 0.5 + (FF*0.5) for alpha SNPs and always 0.5 for beta SNPs, whereas q0 is 0.5 for alpha SNPs and 0.5 - (FF*0.5) for beta SNPs.

Table ST2 provides formulas for q1 and q0 in the various anomalous situations. Most imply applying a correction factor to the standard formulas used for normal pregnancies. When these corrections result in increasing q1 and/or decreasing q0, the respective diagnostic thresholds are spread farther apart and achieving significance becomes more difficult.

Most formulas rely on the apparent fetal fraction, FF, calculated from type-1 SNPs as 2*paternal allele count / total count. All aneuploidies modify FF, either by altering the number of paternal haplotypes, the total allele count, or both. Measuring FF may be impossible for anomalies in which the fetus did not receive a paternal chromosome (e.g. maternal UPDs or monosomy), unless there is some level of mosaicism. But if the panel was suitably designed, it is possible to obtain the real fetal fraction (RFF) from type-1 SNPs located on one or several other chromosomes. Table ST3 provides formulas to calculate q1 and q0 using RFF instead of FF.

For paternal monosomy and paternal UPDs, the fetus carries no maternal haplotype and RHDO is thus not needed (and impossible). However, in mosaic situations it may be interesting to determine the genotype of the normal cells, as these might be predominant in the fetus. The formulas in table ST2 and ST3 can be used for this purpose, although results are unlikely to reach significance when mosaicism is greater than 80%.

Table ST4 provides formulas to obtain FF from RFF and conversely, in the various anomalous situations.

For mosaic anomalies, calculations are heavily affected by the amount of mosaicism m, expressed as aneuploid cells / total cells. This value can be obtained from the regression slopes of FF measured with type-4 versus type-1 SNPs (either alpha or beta SNPs), or from the comparison of FF and RFF, when both are measured experimentally. Table ST5 provides formulas to calculate m in these 3 different manners, for the various anomalies.

All equations are demonstrated in supplementary file 1, and a mosaicism calculator is included for each anomaly.

# Supplementary tables

**Table ST1. Regression slopes for the various anomalies**. The FF calculated with type-4 alpha and type-4 beta SNPs were plotted against FF calculated with type-1 SNPs for a range of FFs. The observed and expected linear regression slopes were computed for the indicated maternal haplotype. For a fetus carrying the opposite maternal haplotype, slope values should be swapped and negated (cf. the two normal cases).

| **Situation** | **Maternal haplotype** | **Observed** | | **Expected** | |
| --- | --- | --- | --- | --- | --- |
|  |  | **Alpha** | **Beta** | **Alpha** | **Beta** |
| Normal, maternal haplotype M1 | M1 | 0.9495 | 0.0055 | 1.0000 | 0.0000 |
| Normal, maternal haplotype M2 | M2 | 0.0055 | -0.9495 | 0.0000 | -1.0000 |
| Discordant twins (33% M1, 67% M2) | 1/3M1 + 2/3M2 | 0.4595 | -0.6144 | 0.3333 | -0.6667 |
|  |  |  |  |  |  |
| Maternal meiosis 1 trisomy | M1 + M2 | 0.5852 | -0.4604 | 0.5000 | -0.5000 |
| Maternal meiosis 1 trisomy, mosaic 50% | M1 + M2 | 0.6900 | -0.2729 | 0.7500 | -0.2500 |
| Maternal meiosis 1 trisomy, mosaic 25% | M1 + M2 | 0.8321 | -0.1440 | 0.8750 | -0.1250 |
| Maternal meiosis 1 trisomy, mosaic 12.5% | M1 + M2 | 0.8908 | -0.1052 | 0.9375 | -0.0625 |
|  |  |  |  |  |  |
| Maternal meiosis 2 trisomy | M1 | 1.3797 | 0.5028 | 1.5000 | 0.5000 |
| Maternal meiosis 2 trisomy, mosaic 50% | M1 | 1.1203 | 0.2513 | 1.2500 | 0.2500 |
|  |  |  |  |  |  |
| Maternal monosomy | M1 | 0.5241 | 0.4356 | 0.5000 | 0.5000 |
|  |  |  |  |  |  |
| Paternal meiosis 1 trisomy | M2 | 0.2678 | -0.7364 | 0.2500 | -0.7500 |
| Paternal meiosis 1 trisomy, mosaic 50% | M2 | 0.1672 | -0.8605 | 0.1250 | -0.8750 |
|  |  |  |  |  |  |
| Paternal meiosis 2 trisomy | M1 | 0.6316 | -0.1970 | 0.7500 | -0.2500 |
| Paternal meiosis 2 trisomy, mosaic 50% | M1 | 0.7725 | -0.1254 | 0.8750 | -0.1250 |
|  |  |  |  |  |  |
| Paternal isodisomy / monosomy | None | 0.4958 | -0.3254 | 0.5000 | -0.5000 |
| Paternal heterodisomy | None | 0.4584 | -0.5346 | 0.5000 | -0.5000 |
| Paternal heterodisomy, mosaic 60% | None | 0.3713 | -0.6430 | 0.3000 | -0.7000 |

**Table ST2: Calculation of significance thresholds using the apparent fetal fraction**

| **Situation** | **q1 alpha** | **q1 beta** | **q0 alpha** | **q0 beta** |
| --- | --- | --- | --- | --- |
| Normal | 0.5 + (FF*0.5) | 0.5 | 0.5 | 0.5 - (FF*0.5) |
| Maternal meiosis 1 trisomy | 0.5 + (FF*0.5) - (FF*0.25*m) | 0.5 - (FF*0.25*m) | 0.5 + (FF*0.25*m) | 0.5 - (FF*0.5) + (FF*0.25*m) |
| Maternal meiosis 2 trisomy | 0.5 + (FF*0.5) + (FF*0.25*m) | 0.5 + (FF*0.25*m) | 0.5 - (FF*0.25*m) | 0.5 - (FF*0.5) - (FF*0.25*m) |
| Maternal monosomy | 0.5 + (FF*0.5) + (FF*0.25*m/(1-m)) | 0.5 + (FF*0.25*m/(1-m)) | 0.5 - (FF*0.25*m/(1-m)) | 0.5 - (FF*0.5) - (FF*0.25*m/(1-m)) |
| Maternal isodisomy | 0.5 + (FF*0.5/(1-m)) | 0.5 + (FF*0.5*m/(1-m)) | 0.5 - (FF*0.5*m/(1-m)) | 0.5 - (FF*0.5/(1-m)) |
| Maternal heterodisomy | 0.5 + (FF*0.5) | 0.5 | 0.5 | 0.5 - (FF*0.5) |
| Paternal trisomy (both) | 0.5 + (FF*0.5) - (FF*0.25*m/(1+m)) | 0.5 - (FF*0.25*m/(1+m)) | 0.5 + (FF*0.25*m/(1+m)) | 0.5 - (FF*0.5) + (FF*0.25*m/(1+m)) |
| Paternal monosomy | 0.5 + (FF*0.5) - (FF*0.25*m) | 0.5 - (FF*0.25*m) | 0.5 + (FF*0.25*m) | 0.5 - (FF*0.5) + (FF*0.25*m) |
| Paternal UPD (both types) | 0.5 + (FF*0.5/(1+m)) | 0.5 - (FF*0.5*m/(1+m)) | 0.5 + (FF*0.5*m/(1+m)) | 0.5 - (FF*0.5/(1+m)) |

FF: Fetal fraction calculated with Type-1 SNPs from the target chromosome (FF = 2*paternal allele/total counts)

m: mosaic fraction (m = normal cells/total cells)

**Table ST3: Calculation of significance thresholds using the real fetal fraction**

| **Situation** | **q1 alpha** | **q1 beta** | **q0 alpha** | **q0 beta** |
| --- | --- | --- | --- | --- |
| Normal | 0.5 + (RFF*0.5) | 0.5 | 0.5 | 0.5 - (RFF*0.5) |
| Maternal meiosis 1 trisomy | 0.5 + RFF*(0.5-0.25*m)  1+(RFF*0.5*m) | 0.5 - RFF*0.25*m__  1+(RFF*0.5*m) | 0.5 + RFF*0.25*m__  1+(RFF*0.5*m) | 0.5 - RFF*(0.5-0.25*m)  1+(RFF*0.5*m)) |
| Maternal meiosis 2 trisomy | 0.5 + RFF*(0.5+0.25*m)  1+(RFF*0.5*m) | 0.5 + RFF*0.25*m__  1+(RFF*0.5*m) | 0.5 - RFF*0.25*m__  1+(RFF*0.5*m) | 0.5 - RFF*(0.5+0.25*m)  1+(RFF*0.5*m)) |
| Maternal monosomy | 0.5 + (RFF*0.5*(1-m)) + (0.25*m)  1-(RFF*0.5*m) | 0.5 + RFF*0.25*m__  1-(RFF*0.5*m) | 0.5 - RFF*0.25*m__  1-(RFF*0.5*m) | 0.5 – (RFF*0.5*(1-m)) + (0.25*m)  1-(RFF*0.5*m) |
| Maternal isodisomy | 0.5 + RFF*0.5 | 0.5 + RFF*0.5*m | 0.5 - RFF*0.5*m | 0.5 - RFF*0.5 |
| Maternal heterodisomy | 0.5 + RFF*0.5*(1-m) | 0.5 | 0.5 | 0.5 - RFF*0.5*(1-m) |
| Paternal trisomy (both) | 0.5 + (RFF*0.5*(1+m)) - (0.25*m)  1+(RFF*0.5*m) | 0.5 - RFF*0.25*m__  1+(RFF*0.5*m) | 0.5 + RFF*0.25*m__  1+(RFF*0.5*m) | 0.5 – (RFF*0.5*(1+m)) - (0.25*m)  1+(RFF*0.5*m) |
| Paternal monosomy | 0.5 + RFF*(0.5-0.25*m)  1-(RFF*0.5*m) | 0.5 - RFF*0.25*m__  1-(RFF*0.5*m) | 0.5 + RFF*0.25*m__  1-(RFF*0.5*m) | 0.5 - RFF*(0.5-0.25*m)  1-(RFF*0.5*m) |
| Paternal UPD (both types) | 0.5 + RFF*0.5 | 0.5 - RFF*0.5*m | 0.5 + RFF*0.5*m | 0.5 - RFF*0.5 |

RFF: Fetal fraction calculated with Type-1 SNPs for one or more different chromosome(s) (RFF = 2*paternal allele/total counts)

m: mosaic fraction (m = normal cells/total cells)

**Table ST4: Conversions between apparent and real fetal fraction**

| **Situation** | **Apparent FF from real FF** | **Real FF from apparent FF** |
| --- | --- | --- |
| Maternal meiosis 1 trisomy | RFF/(1+(RFF*0.5*m) | FF/(1-(FF*0.5*m) |
| Maternal meiosis 2 trisomy | RFF/(1+(RFF*0.5*m) | FF/(1-(FF*0.5*m) |
| Maternal monosomy | (RFF*(1-m)) / (1-(RFF*0.5*m)) | FF/(1-m+(FF*0.5*m)) |
| Maternal isodisomy | RFF*(1-m) | FF/(1- m) |
| Maternal heterodisomy | RFF*(1-m) | FF/(1- m) |
| Paternal trisomy (both) | (RFF*(1+m)) / (1+(RFF*0.5*m)) | FF / (1+m-(FF*0.5*m)) |
| Paternal monosomy | RFF/(1-(RFF*0.5*m)) | FF/(1+(FF*0.5*m)) |
| Paternal UPD (both types) | RFF*(1+m) | FF/(1+m) |

FF: Apparent fetal fraction, calculated with Type-1 SNPs from the target chromosome

RFF: Real fetal fraction, calculated with Type-1 SNPs from one or more different chromosomes

m: mosaic fraction (m = normal cells / total cells)

**Table ST5: Estimation of mosaic fraction (3 methods)**

| **Situation** | **From FF and RFF** | **From alpha slope** | **From beta slope** |
| --- | --- | --- | --- |
| Maternal meiosis 1 trisomy | 2/FF - 2/RFF | 2*(1-SA) | -2*SB |
| Maternal meiosis 2 trisomy | 2/FF - 2/RFF | 2*(SA-1) | 2*SB |
| Maternal monosomy | (FF-RFF) / ((FF*RFF*0.5)-RFF) | 2*(1-SA) | 2*SB |
| Maternal isodisomy | (RFF-FF) / RFF | Invariant | SB |
| Maternal heterodisomy | (RFF-FF) / RFF | 1-SA | Invariant |
| Paternal trisomy (both) | (FF-RFF) / (RFF*(1-(FF*0.5))) | 4*(1-SA) | -4*SB |
| Paternal monosomy | (FF-RFF) / (RFF*FF*0.5) | 2*(1-SA) | -2*SB |
| Paternal UPD (both types) | (FF/RFF)-1 | 2*(1-SA) | -2*SB |

FF: Apparent fetal fraction, calculated with Type-1 SNPs from the target chromosome.

RFF: Real fetal fraction, calculated with Type-1 SNPs from one or more different chromosomes

SA: Regression slope for alpha SNPs

SB: Regression slope for beta SNPs

# Legends for supplementary figures

**Supplementary figure 1. Incorrect RHDO result in a suspected case of vanishing twin**

**A**: In the prenatal sample, the cumulative allelic balance for type-4 SNP (diamonds) downstream of the mutation clearly indicates that the presumed singleton fetus inherited maternal haplotype M2 (orange curve: diagnostic threshold for M2).

**B**: Composite postnatal sample created by mixing DNA from the newborn child and maternal DNA in the same proportions as in the prenatal sample. Here, the allelic balance indicates haplotype M1 (blue curve: diagnostic threshold for M1), which is the correct result, verified with DNA from the newborn child.

**C**: Frequency of paternal-specific alleles for type-3 SNPs in ccfDNA. Both paternal haplotypes are present, albeit at different frequencies (approximately 6.7% and 3.2%). Genomic positions are indicated on the X-axis, allelic frequency on the Y-axis. The overlapping whisker boxes on the right are mean ±SD of allelic frequencies for type-2 SNPs (bottom box, background) and type-1 SNPs (upper box). Whiskers: ±2 SD.

**D**: Similar results with a different chromosome, indicating that the most likely explanation is a twin pregnancy. Post-natal analysis confirmed that the newborn child carried paternal haplotype P2 and maternal haplotype M1 at the GCK locus and paternal haplotype P1 at the GLDN locus. Analysis of the allegedly vanished twin was not possible.

**E, F**: Regression analysis of type-4 SNPs versus type-1 SNPs in the *GCK* and *GLDN* genes. The abnormal slope values (normal: alpha = 0, beta = -1) are compatible with twin fetuses contributing uneven amounts of ccfDNA.

**Supplementary figure 2. Experimental design**

1) One female and six male patients were genotyped with a panel of SNPs targeting 4 loci on the X chromosome.

2) The most informative combinations were selected, and composite samples were created by mixing DNA from two male patients (3 patients for trisomies resulting from non-disjunction in meiosis 1).

3) Composite samples were assayed in the same manner as genuine ccfDNA samples, to verify that the expected allelic balance was obtained.

4) To simulate prenatal samples, a composite sample representing the fetus was mixed with a genetically matched composite sample representing the mother, generally aiming for a proportion of 20%. These samples were assayed as above.

5) To simulate mosaicism, an abnormal composite sample and a matching normal sample were mixed with a “maternal” sample and assayed as above.

6) Sequencing data were analyzed with our standard NIPD-M pipeline (results not shown).

7) FF signatures were produced for all samples.

8) To reduce FF, sequencing data from composite samples were mixed in various proportions with sequencing data from the corresponding maternal sample. FFs calculated with type-4 alpha and beta SNPs were plotted against the FFs determined with type-1 SNPs for regression analysis.

**Supplementary figure 3. Verification of allelic frequencies**

Allelic balances in composite samples, prior to mixing with maternal samples.

**Supplementary figures 4 to 19**

FF signatures for the various anomalies. To reduce FF, sequencing data from the simulated prenatal sample were diluted with data from the corresponding “maternal” sample. The resulting FF is indicated under each graph.

**Supplementary figure 20. FF signatures for chromosome X**

**Top**: Family with a maternal duplication in the DMD gene, transmitted to the first daughter (#1). Three NIPD-M tests were requested around 11 weeks since LMP. The mutation (maternal haplotype M1) was present in all 3 fetuses, however #2 and #3 were male, whereas #4 was female.

**Bottom**: FF signatures for the 3 pregnancies (SNPs located inside the duplication were excluded from this analysis). With male fetuses, the expected signature is that of a maternal monosomy (FF calculated from 4 loci on chromosome Y). For female fetuses, the signature is the same as that of an autosomal chromosome, except that there are no type-3 SNPs (paternal SNPs are considered Type-1 and used to calculate FF).

**Supplementary figure 21. Retrospective analyses**

**A**: FF signatures were retrospectively generated from our clinical cases for which suitable data was available. FF calculated with type-3 SNPs (blue symbols) and type-4 SNPs (magenta) was normalized to that of type-1 SNPs (dotted line, 100%). Triangles represent the transmitted haplotype, circles the haplotype absent in the fetus. Error bars are SD between individual SNPs. The blue area is the background threshold for type-3 SNPs: mean of type-2 SNPs normalized frequency +1 SD. The pink area is used for type-4 SNPs: average SD for “present” alleles from type-1, type-3 and type-4 SNPs. The amber area is the normal range for haplotypes that are present in the fetus: 100% +/-1 SD of type-1 SNP frequencies. Data points are missing when there were less than 3 SNPs of a given subtype, or when haplotypes were phased in only 1 parent (using the grandparents, as no prior child was available). Suspicious data points are labelled with letters corresponding to further analyses illustrated in panels B through F and figure S1.

**B**: Regression analysis of type-4 SNPs for the HBB locus. The pattern of slopes suggests a maternal meiosis 2 trisomy of chromosome 11, probably with some level of mosaicism (expected values for a homogeneous trisomy: alpha = 1.5, beta = 0.5).

**C**: Regression analysis of type-4 SNPs for the RYR1 locus. Normal slopes, maternal haplotype M1.

**D**: Regression analysis of type-4 SNPs for the GCK test locus. The pattern of slopes (normal for alpha, -0.4 for beta) does not correspond to any anomaly.

**E**: Regression analysis of type-4 SNPs for the GCK locus. The pattern of slopes is suggestive of a maternal meiosis 1 trisomy of chromosome 7, in a 50% mosaic state (expected slopes: alpha = 0.25 and beta = -0.75).

**F**: Analysis of individual type-3 SNPs for the GLDN locus. Although haplotype P2 appears to be present, virtually all SNP frequencies fall within 1 SD of the background, confirming that it was simply an unusually noisy experiment. Whisker boxes are mean +/- 1 SD. Whiskers are +/-2 SD.
