# Supplementary material for: Fetal Fraction Signatures: A Quality Control Tool to Detect Potentially Confounding Situations in NonInvasive Prenatal Diagnosis of Monogenic Conditions
Source: Clin Genet. 2025 Dec 9;109(5):869–75. doi: 10.1111/cge.70121 (PMC13066760; doi:10.1111/cge.70121)
Supplement: Supplementary file 3 — Data S3: cge70121‐sup‐0003‐Figures.pptx. [file CGE-109-869-s002.pptx]

## Slide 1
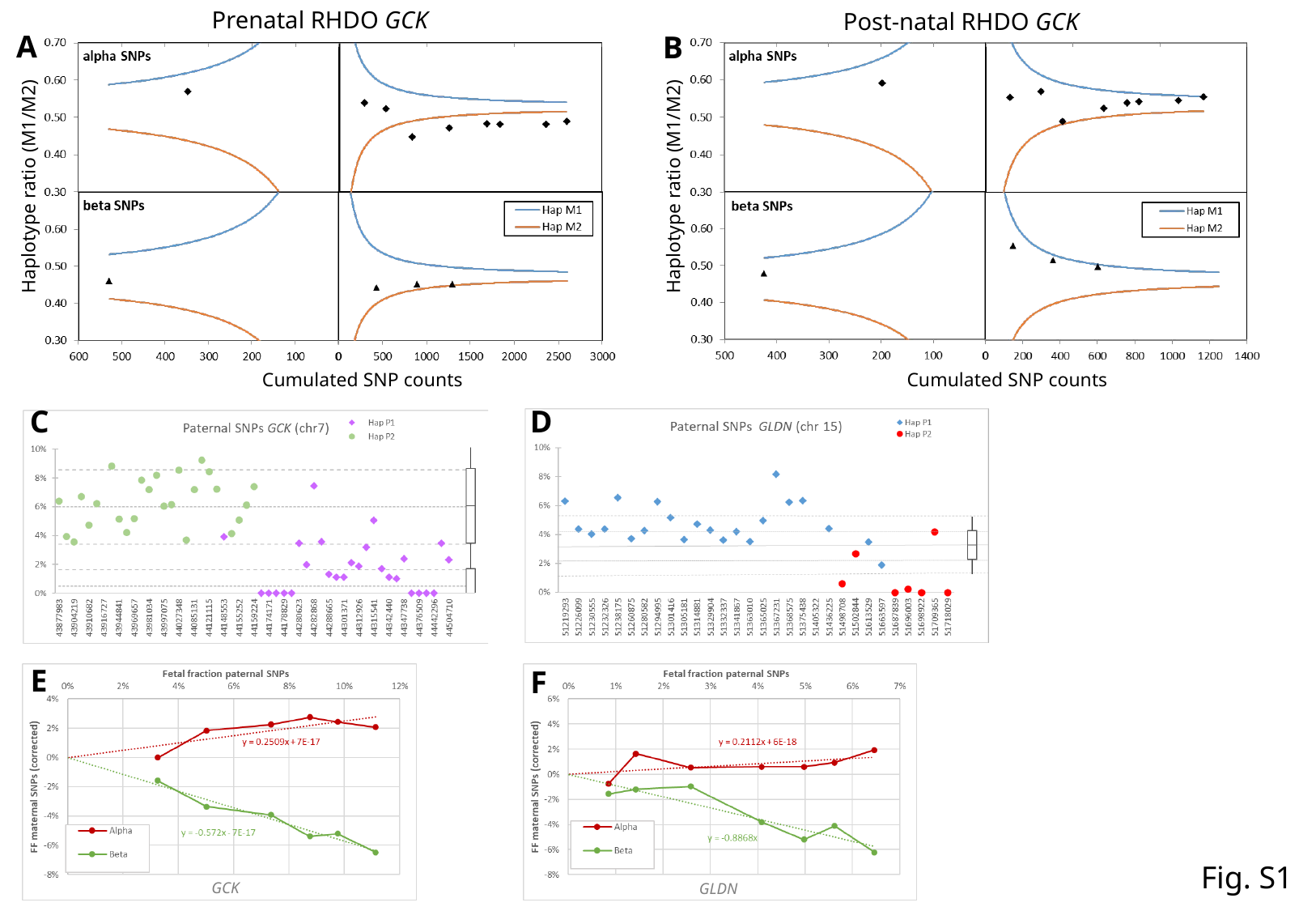

Prenatal RHDO GCK
A
Haplotype ratio (M1/M2)
Cumulated SNP counts
Post-natal RHDO GCK
B
Haplotype ratio (M1/M2)
Cumulated SNP counts
C
D
E
F
Fig. S1
GCK
GLDN

## Slide 2
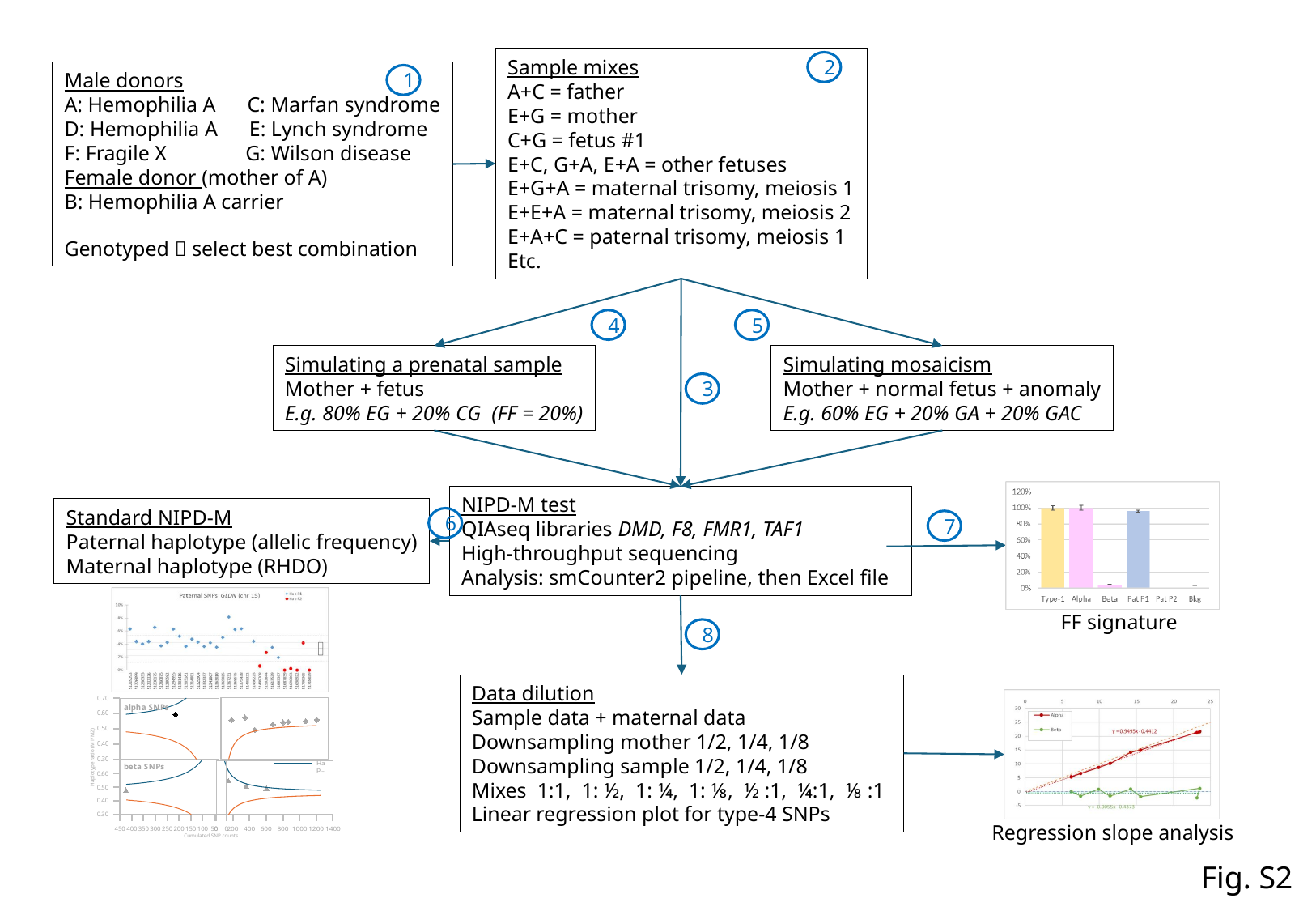

Sample mixes
A+C = father
E+G = mother
C+G = fetus #1
E+C, G+A, E+A = other fetuses
E+G+A = maternal trisomy, meiosis 1
E+E+A = maternal trisomy, meiosis 2
E+A+C = paternal trisomy, meiosis 1
Etc.
Male donors
A: Hemophilia A C: Marfan syndrome
D: Hemophilia A E: Lynch syndrome
F: Fragile X G: Wilson disease
Female donor (mother of A)
B: Hemophilia A carrier
Genotyped  select best combination
4
5
Simulating a prenatal sample
Mother + fetus
E.g. 80% EG + 20% CG (FF = 20%)
Simulating mosaicism
Mother + normal fetus + anomaly
E.g. 60% EG + 20% GA + 20% GAC
3
NIPD-M test
QIAseq libraries DMD, F8, FMR1, TAF1
High-throughput sequencing
Analysis: smCounter2 pipeline, then Excel file
Standard NIPD-M
Paternal haplotype (allelic frequency)
Maternal haplotype (RHDO)
8
Data dilution
Sample data + maternal data
Downsampling mother 1/2, 1/4, 1/8
Downsampling sample 1/2, 1/4, 1/8
Mixes 1:1, 1: ½, 1: ¼, 1: ⅛, ½ :1, ¼:1, ⅛ :1
Linear regression plot for type-4 SNPs
### Chart
| Category | | | |
|---|---|---|---|
### Chart: alpha SNPs
| Category | | | |
|---|---|---|---|Haplotype ratio (M1/M2)
### Chart: beta SNPs
| Category | | | |
|---|---|---|---|
### Chart
| Category | | | |
|---|---|---|---|Cumulated SNP counts
2
1
6
7
FF signature
Regression slope analysis
Fig. S2

## Slide 3
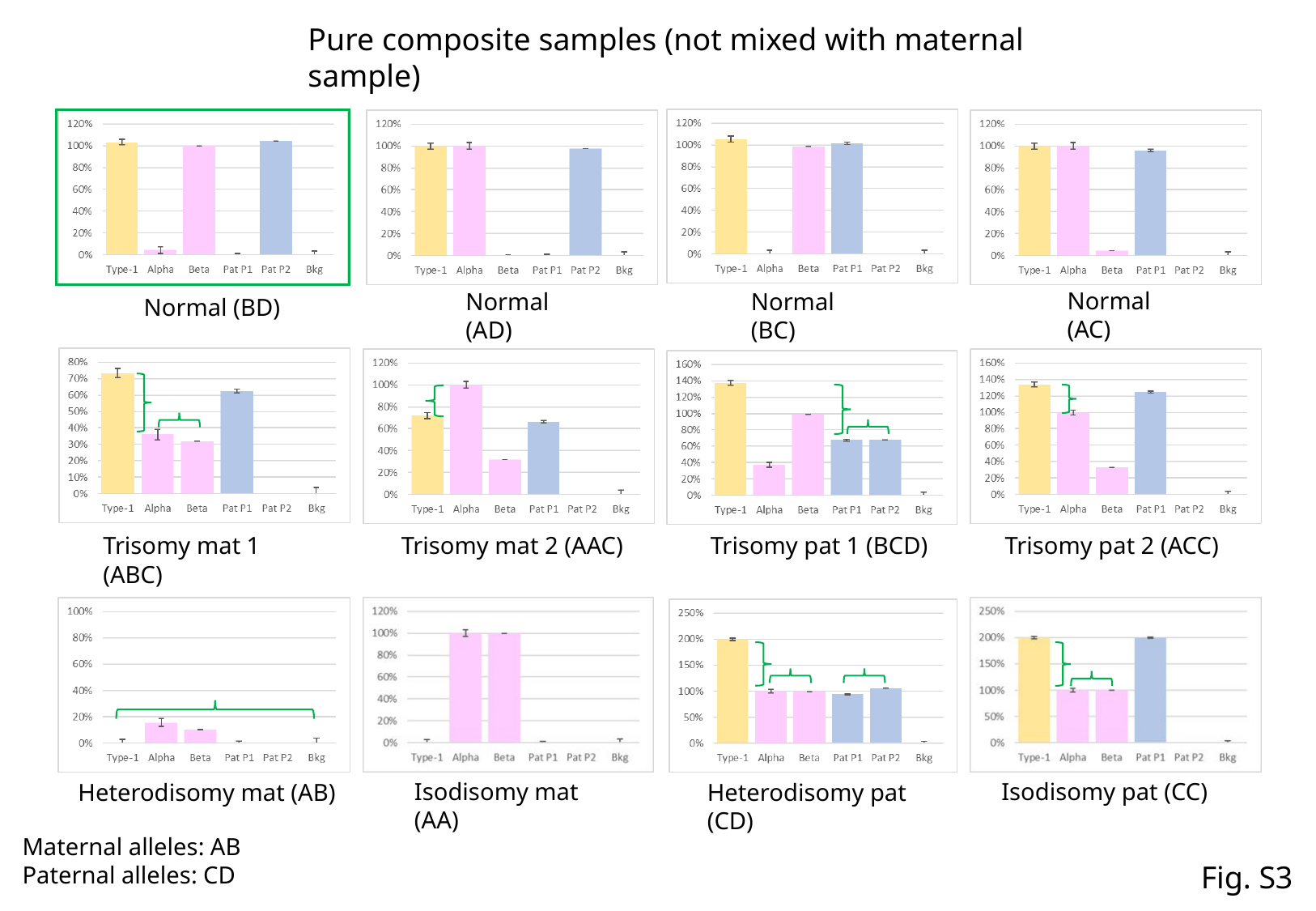

Pure composite samples (not mixed with maternal sample)
Normal (AC)
Normal (AD)
Normal (BC)
Normal (BD)
Trisomy mat 1 (ABC)
Trisomy mat 2 (AAC)
Trisomy pat 1 (BCD)
Trisomy pat 2 (ACC)
Isodisomy mat (AA)
Isodisomy pat (CC)
Heterodisomy mat (AB)
Heterodisomy pat (CD)
Maternal alleles: AB
Paternal alleles: CD
Fig. S3

## Slide 4
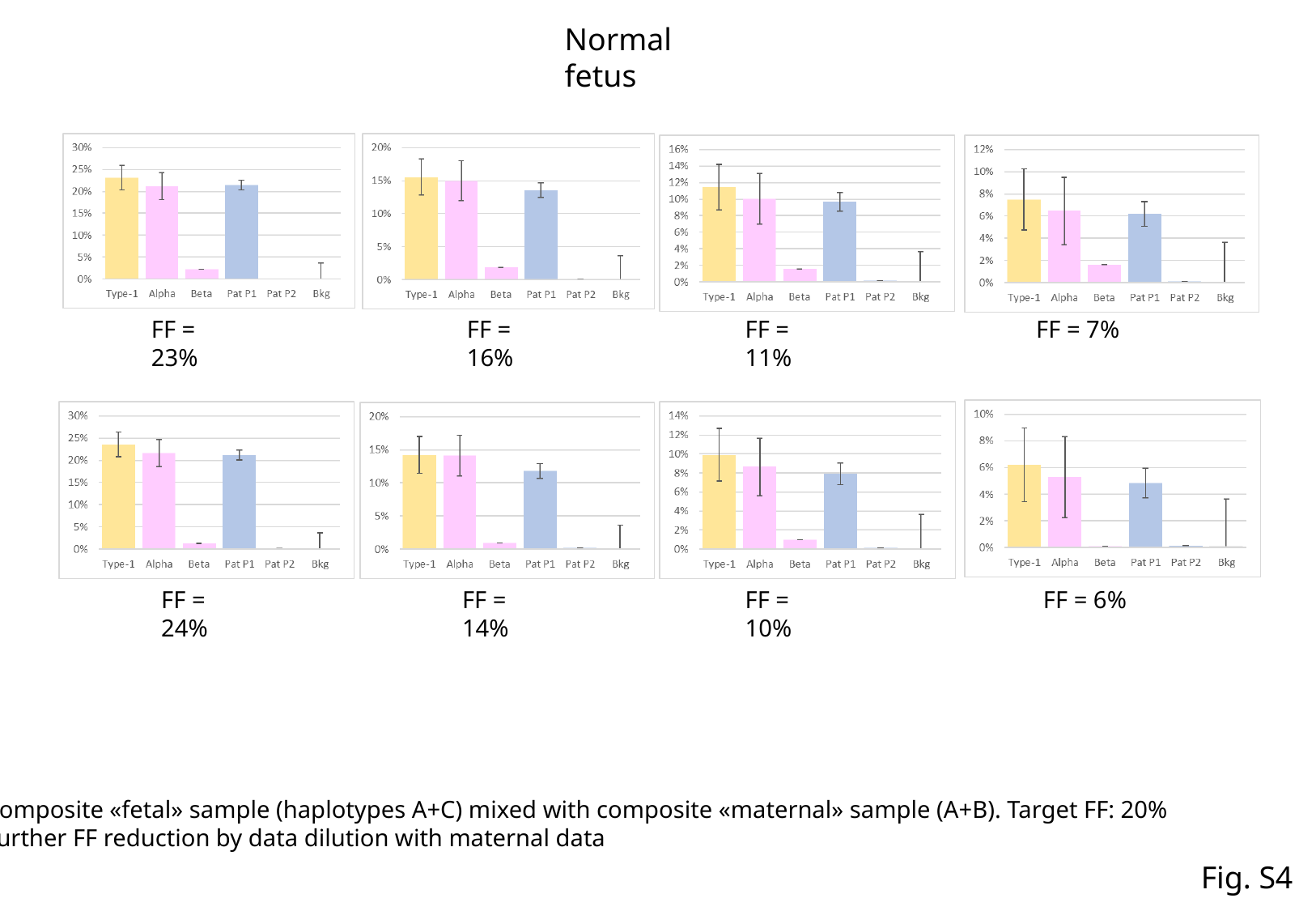

Normal fetus
FF = 23%
FF = 16%
FF = 11%
FF = 7%
FF = 24%
FF = 14%
FF = 10%
FF = 6%
Composite «fetal» sample (haplotypes A+C) mixed with composite «maternal» sample (A+B). Target FF: 20%
Further FF reduction by data dilution with maternal data
Fig. S4

## Slide 5
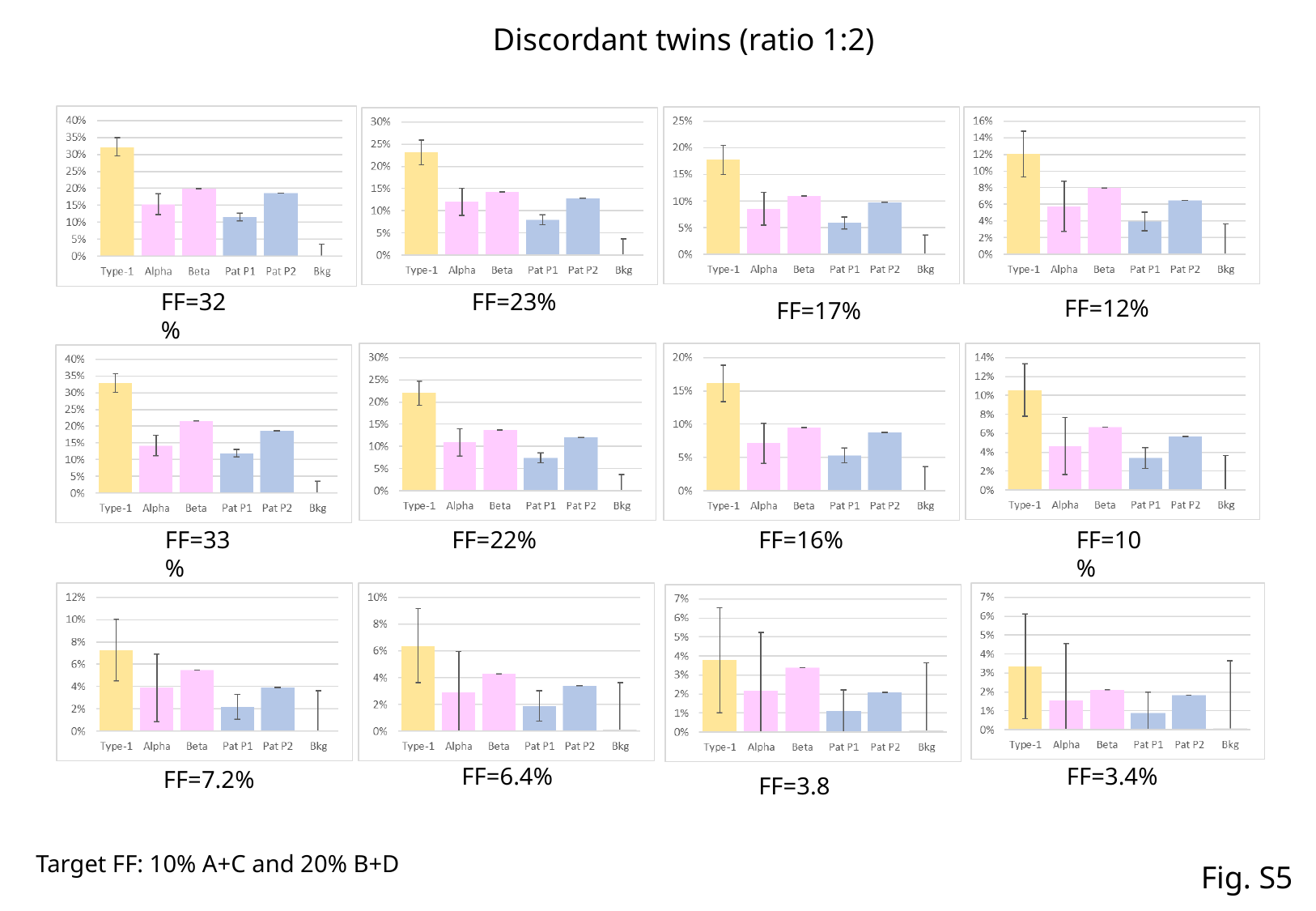

Discordant twins (ratio 1:2)
FF=32%
FF=23%
FF=12%
FF=17%
FF=33%
FF=22%
FF=16%
FF=10%
FF=3.4%
FF=6.4%
FF=7.2%
FF=3.8
Target FF: 10% A+C and 20% B+D
Fig. S5

## Slide 6
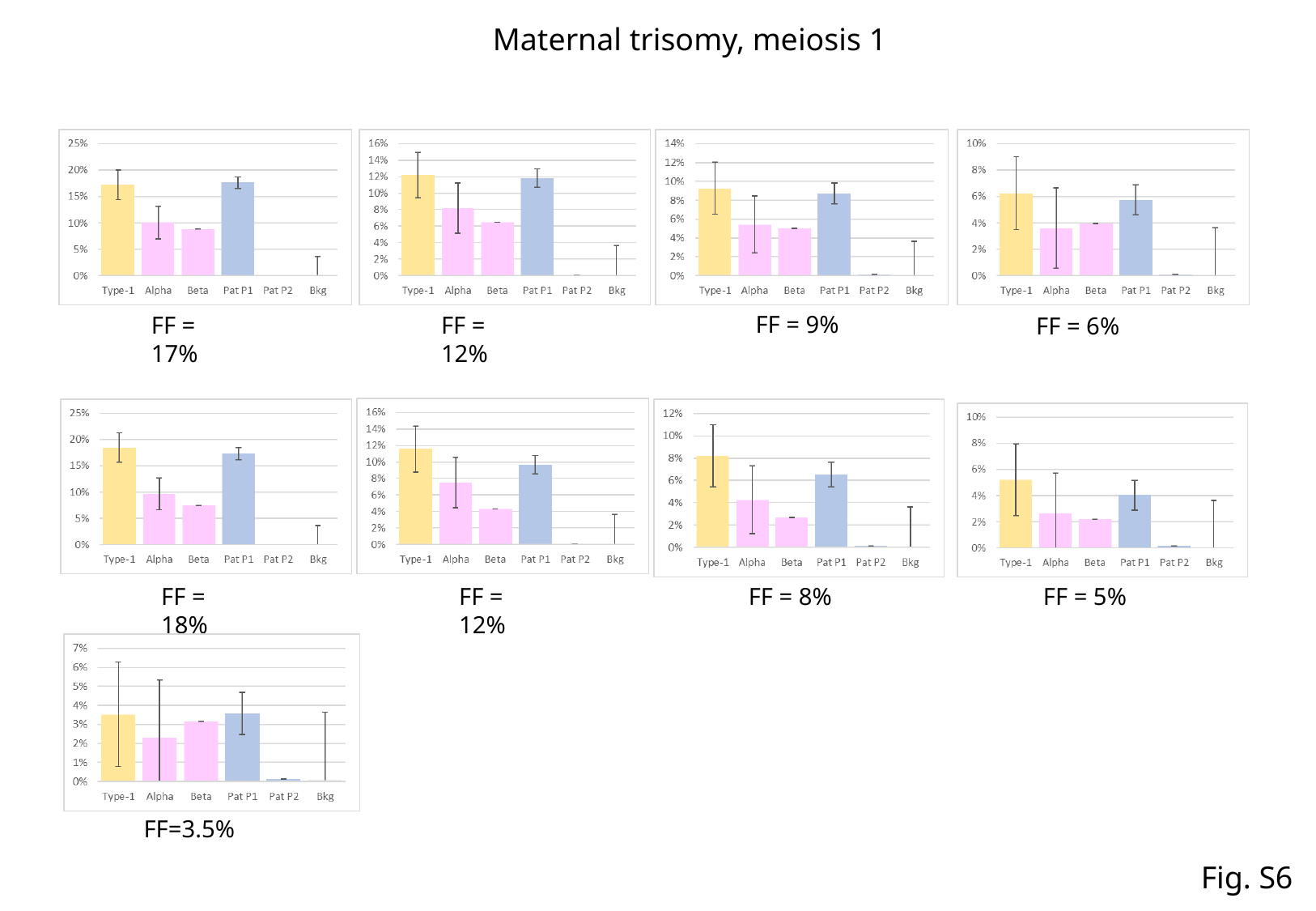

Maternal trisomy, meiosis 1
FF = 9%
FF = 17%
FF = 12%
FF = 6%
FF = 18%
FF = 12%
FF = 8%
FF = 5%
FF=3.5%
Fig. S6

## Slide 7
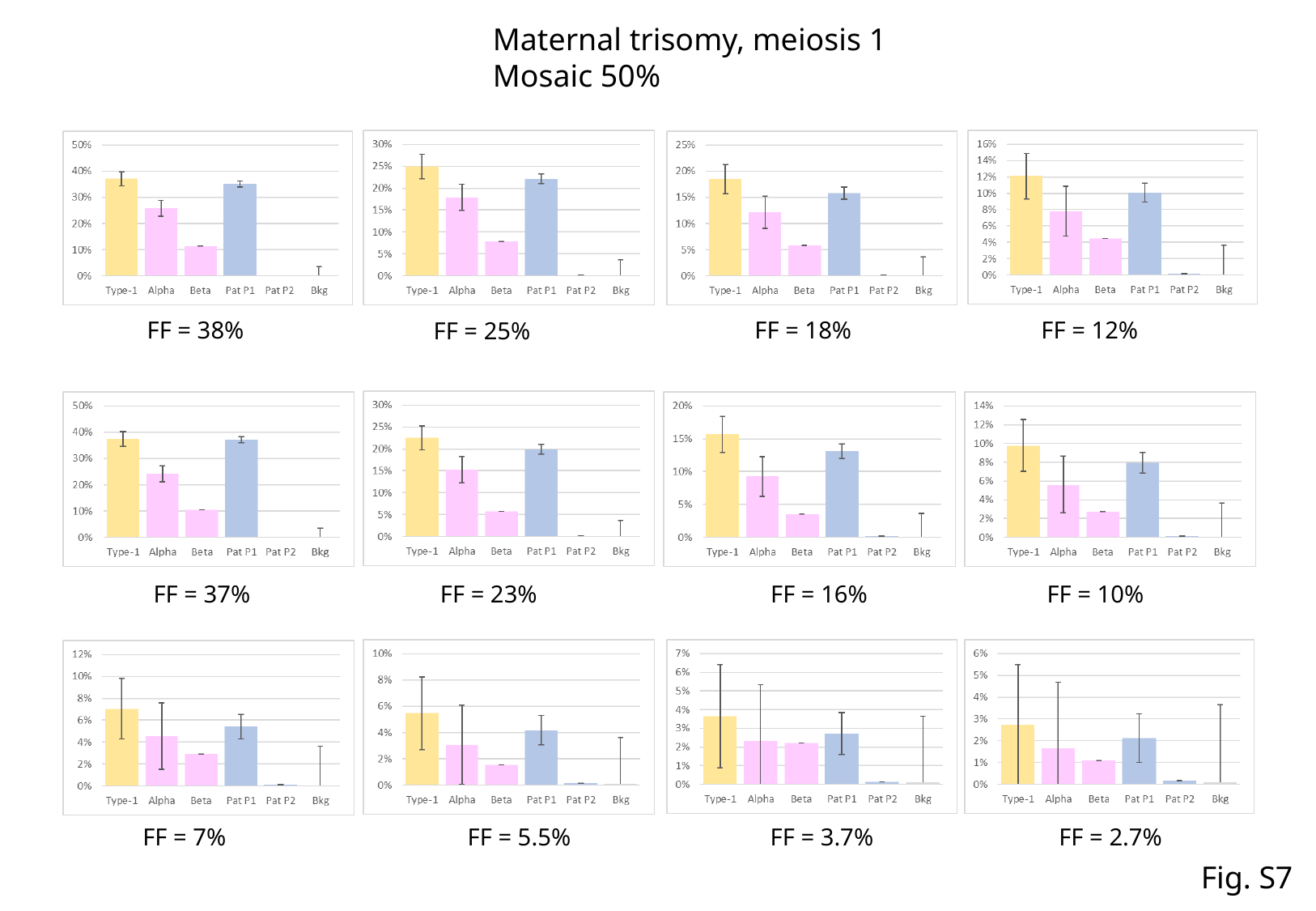

Maternal trisomy, meiosis 1
Mosaic 50%
FF = 38%
FF = 18%
FF = 12%
FF = 25%
FF = 37%
FF = 23%
FF = 16%
FF = 10%
FF = 7%
FF = 5.5%
FF = 3.7%
FF = 2.7%
Fig. S7

## Slide 8
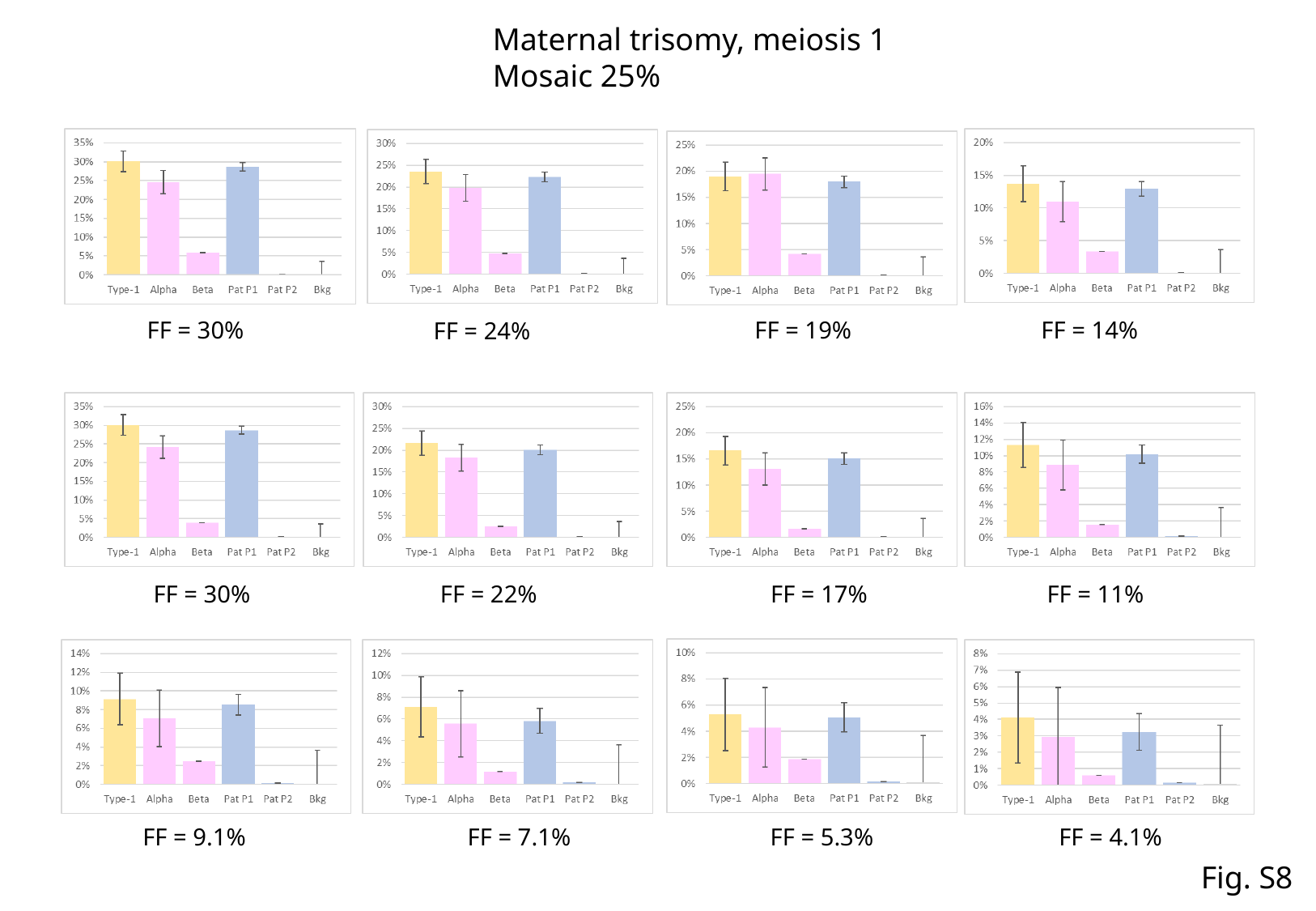

Maternal trisomy, meiosis 1
Mosaic 25%
FF = 30%
FF = 19%
FF = 14%
FF = 24%
FF = 30%
FF = 22%
FF = 17%
FF = 11%
FF = 9.1%
FF = 7.1%
FF = 5.3%
FF = 4.1%
Fig. S8

## Slide 9
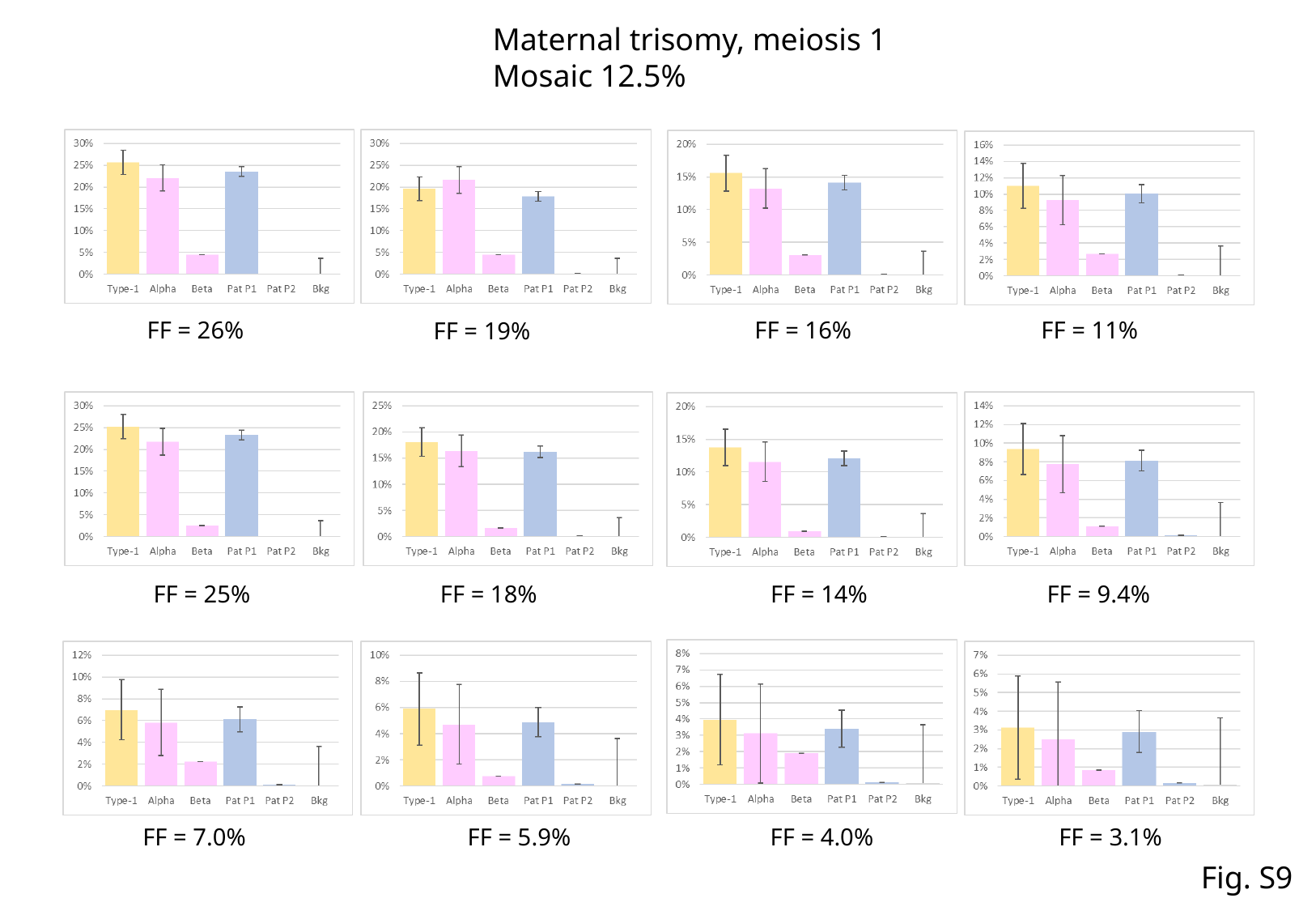

Maternal trisomy, meiosis 1
Mosaic 12.5%
FF = 26%
FF = 16%
FF = 11%
FF = 19%
FF = 25%
FF = 18%
FF = 14%
FF = 9.4%
FF = 7.0%
FF = 5.9%
FF = 4.0%
FF = 3.1%
Fig. S9

## Slide 10
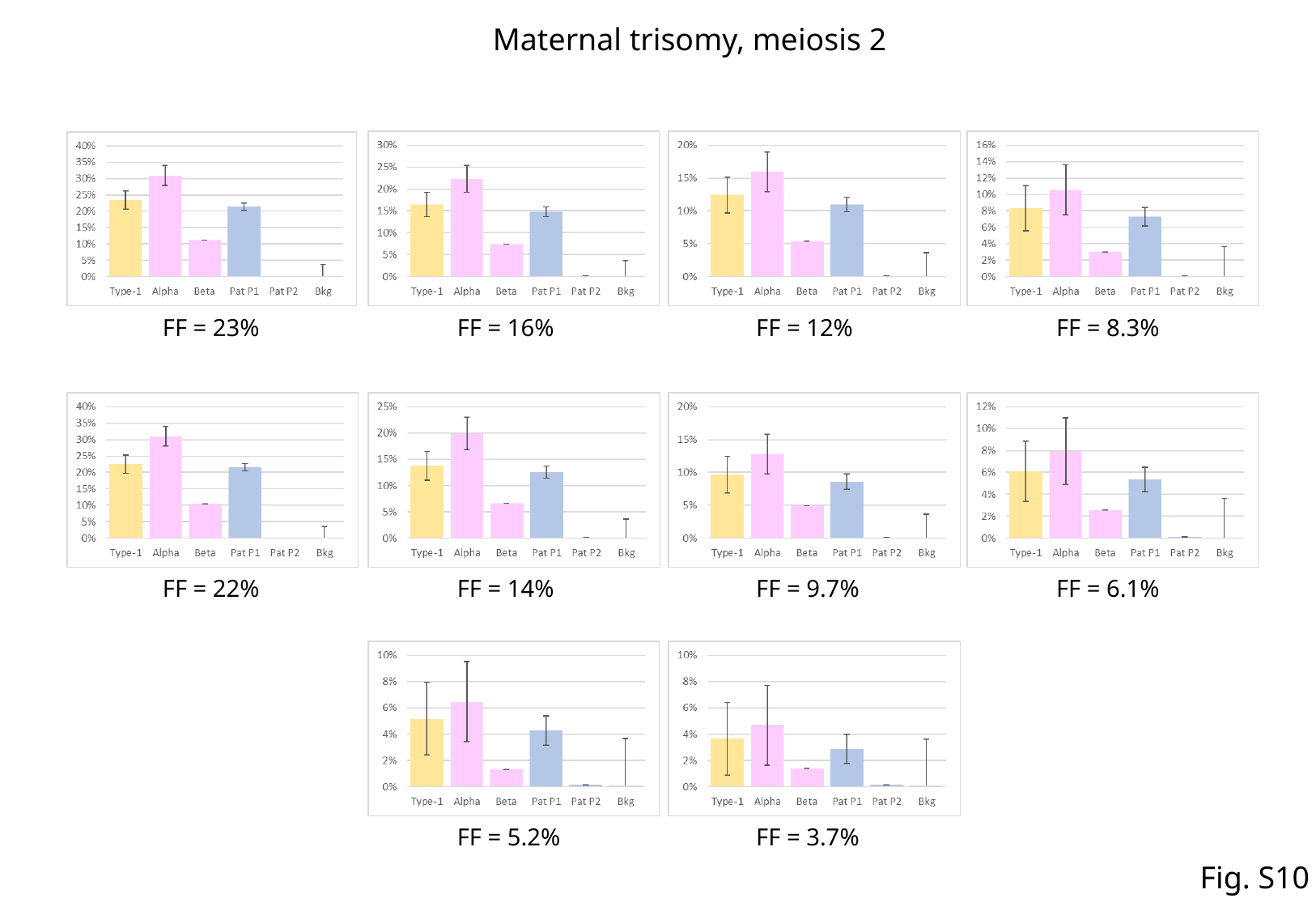

Maternal trisomy, meiosis 2
FF = 23%
FF = 16%
FF = 12%
FF = 8.3%
FF = 22%
FF = 14%
FF = 9.7%
FF = 6.1%
FF = 5.2%
FF = 3.7%
Fig. S10

## Slide 11
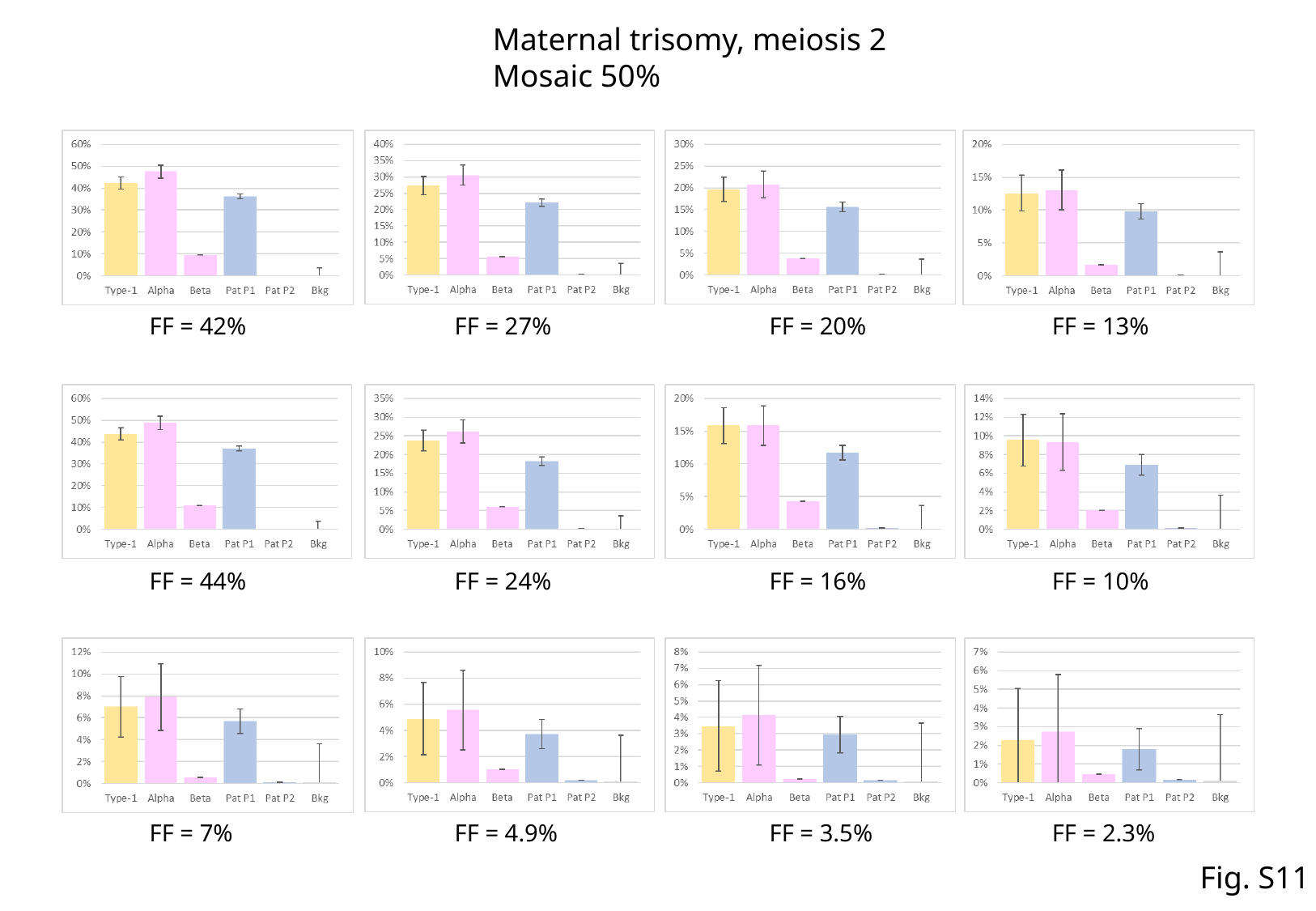

Maternal trisomy, meiosis 2
Mosaic 50%
FF = 42%
FF = 27%
FF = 20%
FF = 13%
FF = 44%
FF = 24%
FF = 16%
FF = 10%
FF = 7%
FF = 4.9%
FF = 3.5%
FF = 2.3%
Fig. S11

## Slide 12
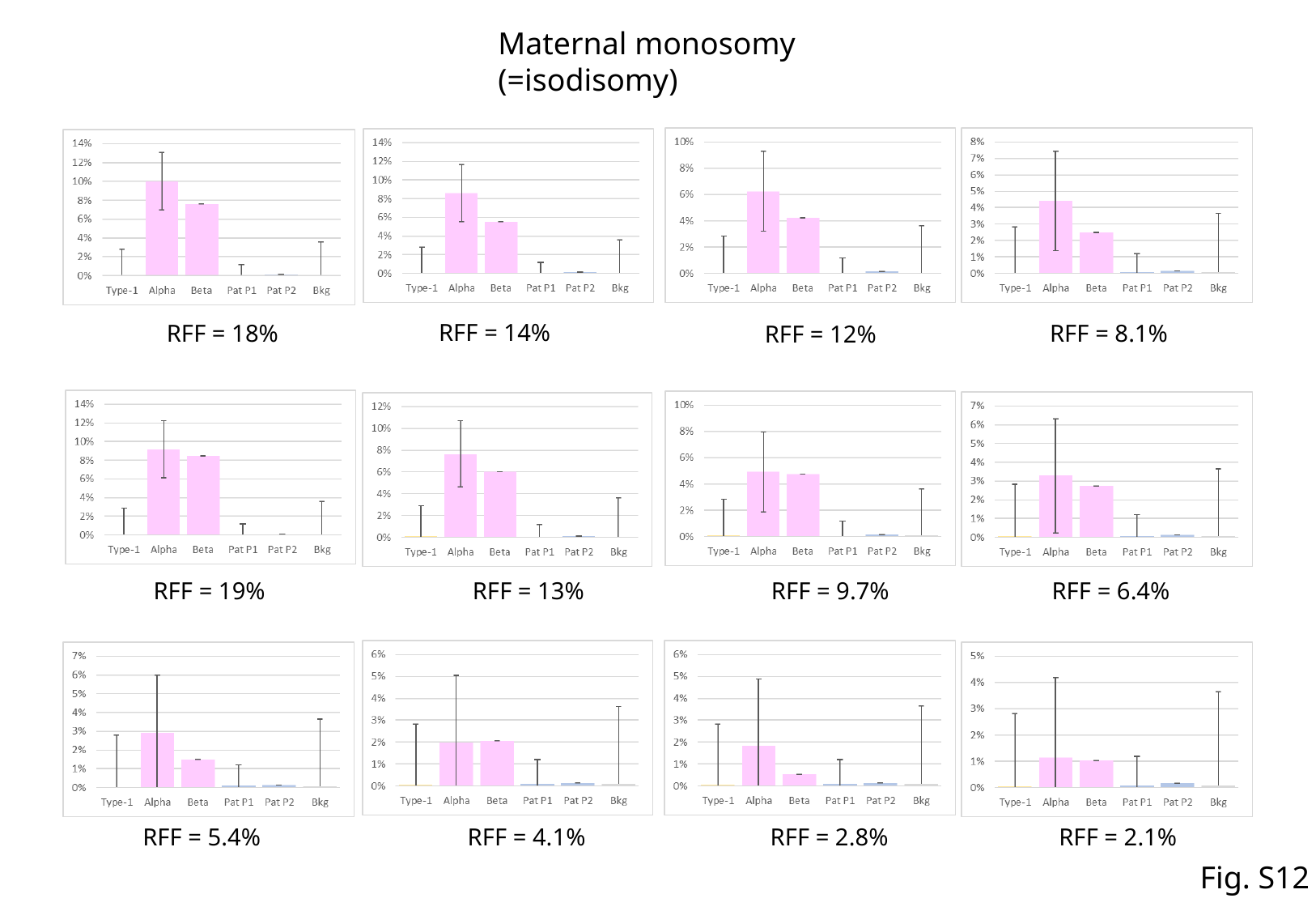

Maternal monosomy (=isodisomy)
RFF = 14%
RFF = 18%
RFF = 8.1%
RFF = 12%
RFF = 19%
RFF = 13%
RFF = 9.7%
RFF = 6.4%
RFF = 5.4%
RFF = 4.1%
RFF = 2.8%
RFF = 2.1%
Fig. S12

## Slide 13
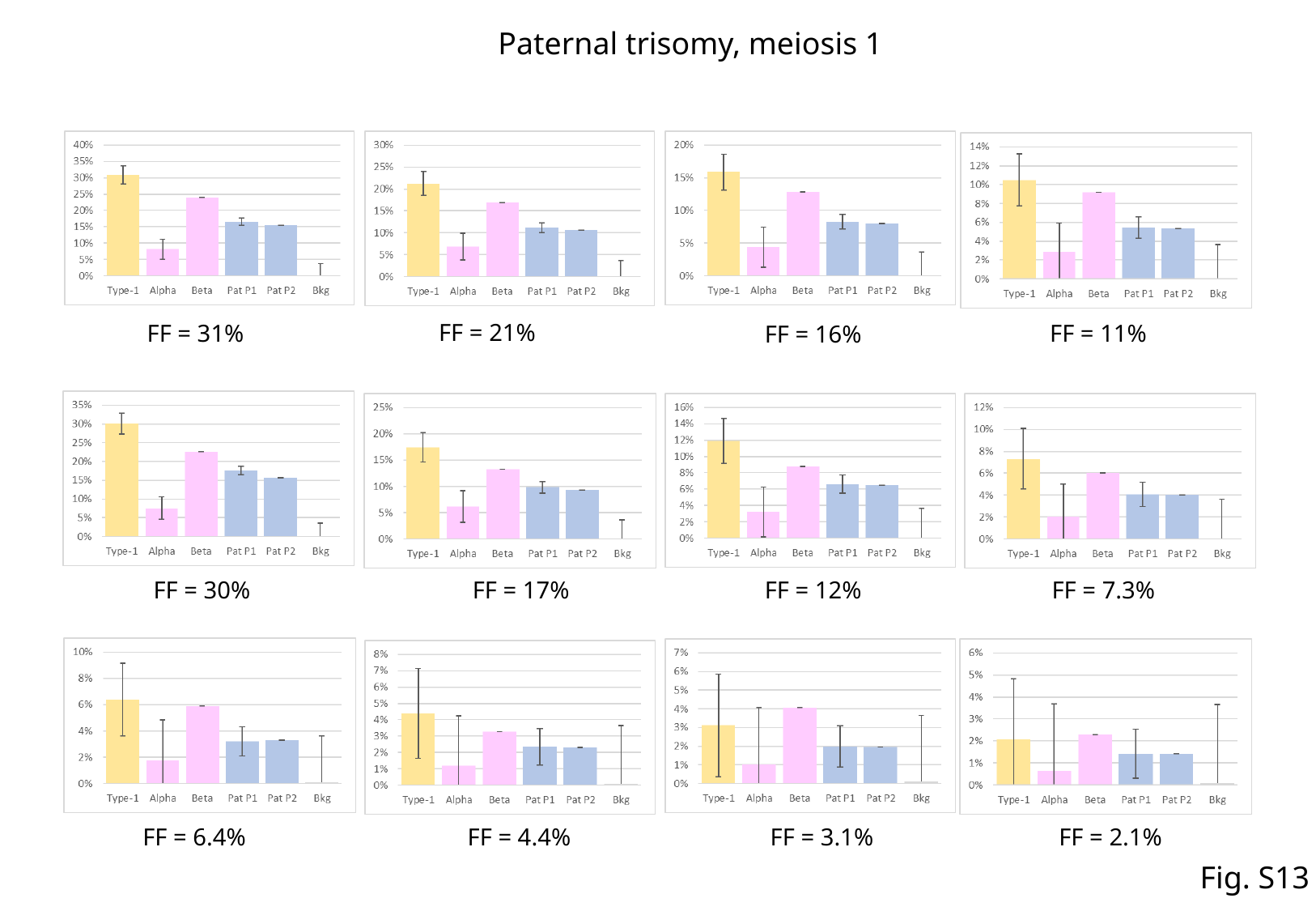

Paternal trisomy, meiosis 1
FF = 21%
FF = 31%
FF = 11%
FF = 16%
FF = 30%
FF = 17%
FF = 12%
FF = 7.3%
FF = 6.4%
FF = 4.4%
FF = 3.1%
FF = 2.1%
Fig. S13

## Slide 14
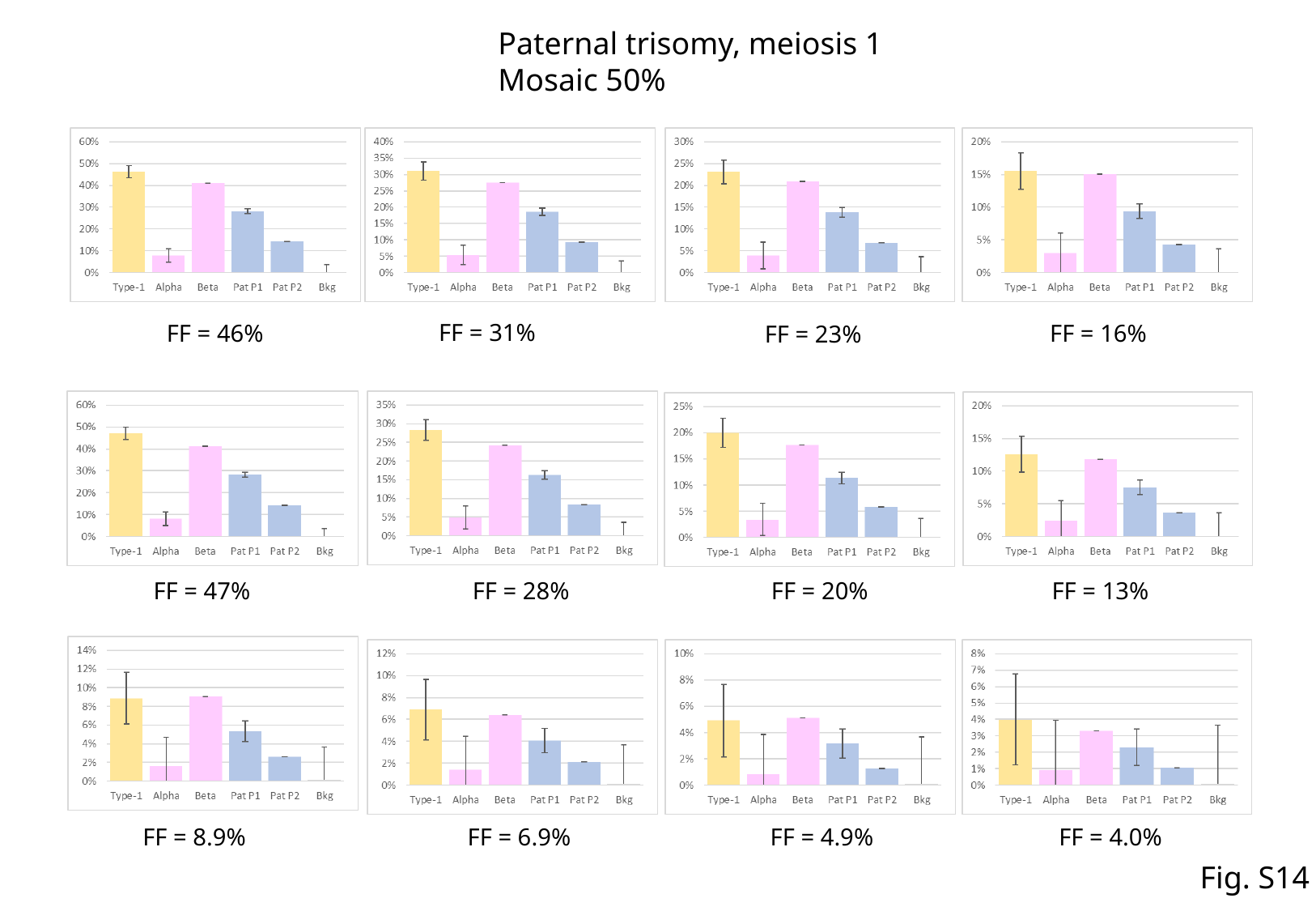

Paternal trisomy, meiosis 1
Mosaic 50%
FF = 31%
FF = 46%
FF = 16%
FF = 23%
FF = 47%
FF = 28%
FF = 20%
FF = 13%
FF = 8.9%
FF = 6.9%
FF = 4.9%
FF = 4.0%
Fig. S14

## Slide 15
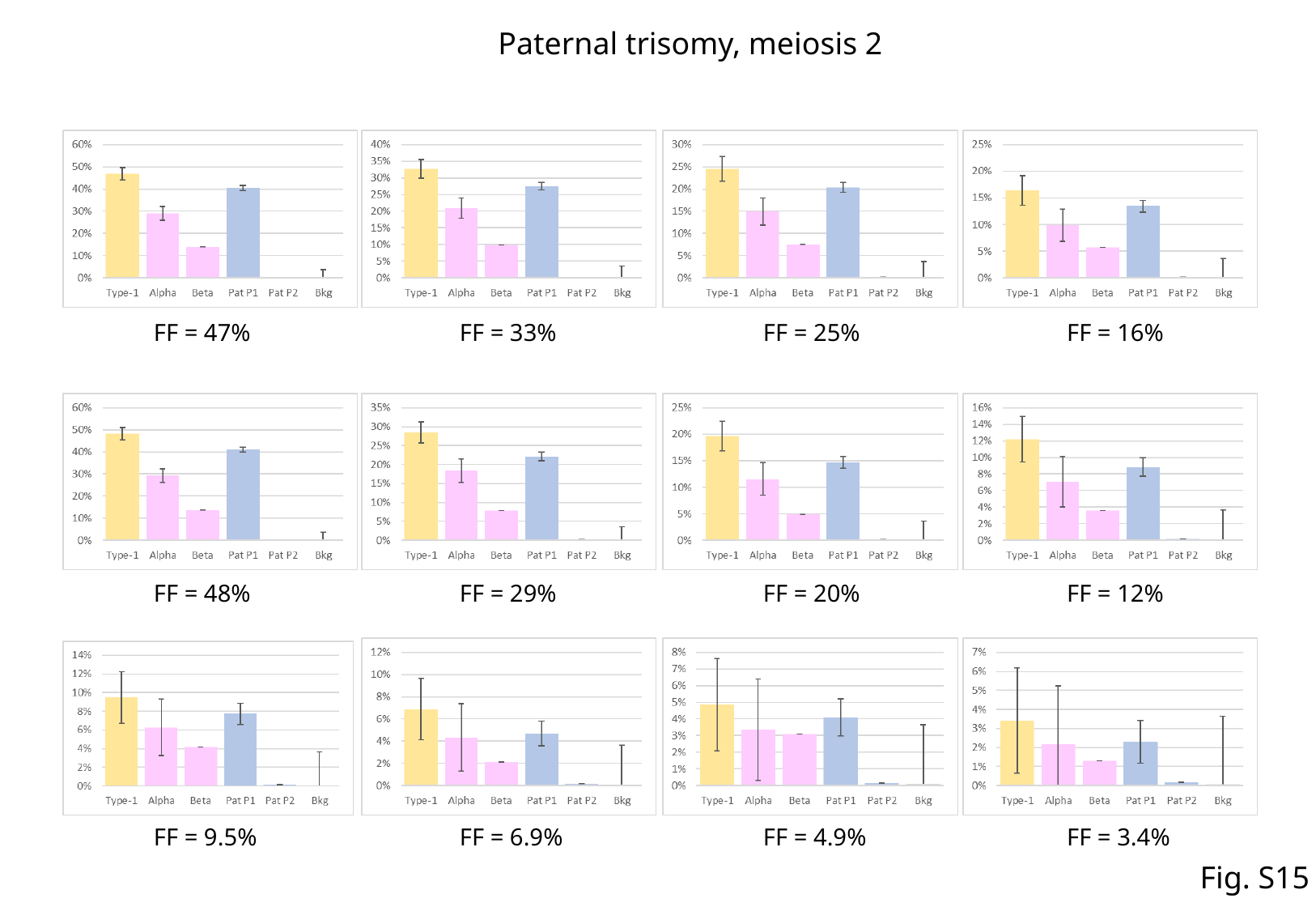

Paternal trisomy, meiosis 2
FF = 47%
FF = 33%
FF = 25%
FF = 16%
FF = 48%
FF = 29%
FF = 20%
FF = 12%
FF = 9.5%
FF = 6.9%
FF = 4.9%
FF = 3.4%
Fig. S15

## Slide 16
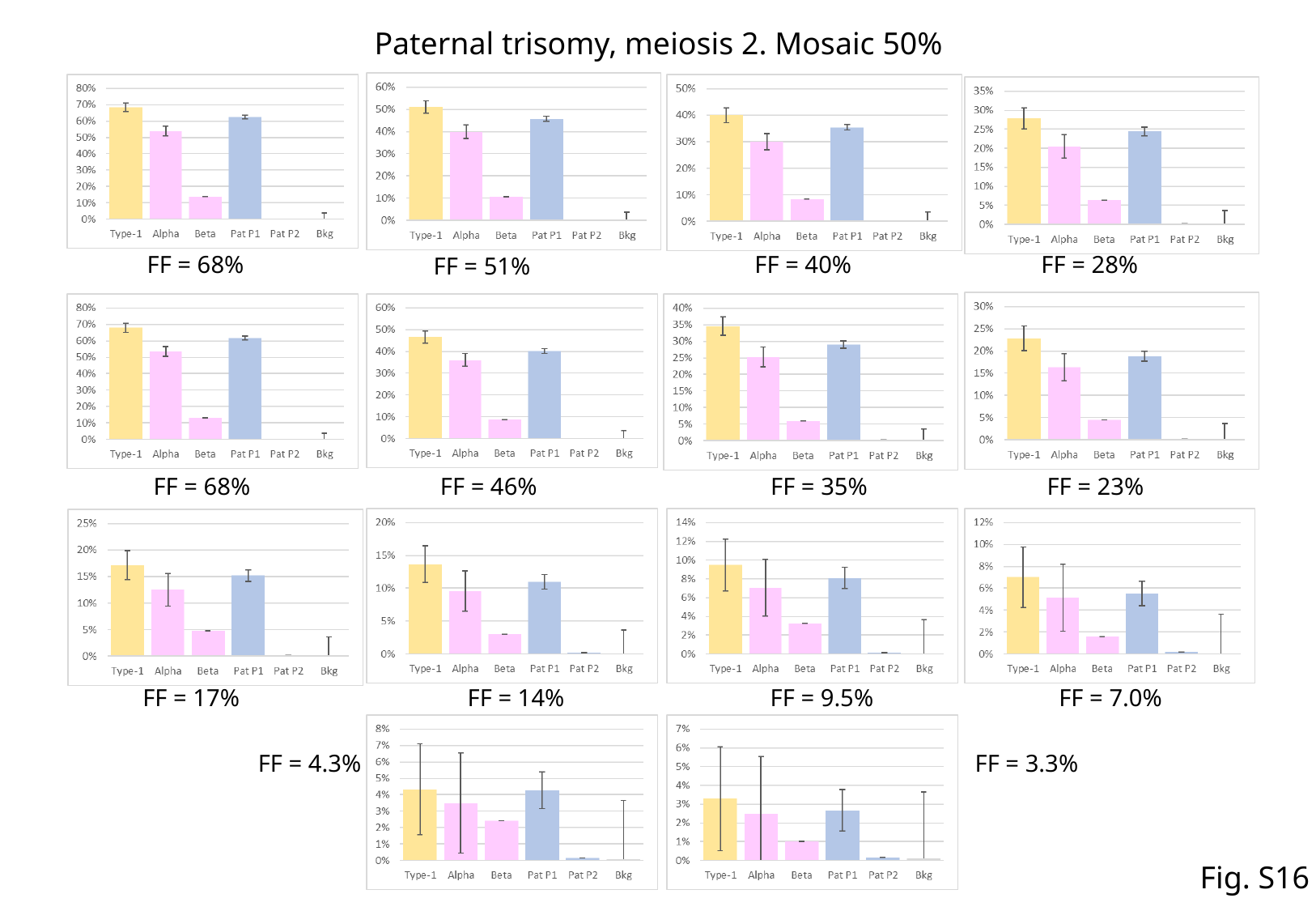

Paternal trisomy, meiosis 2. Mosaic 50%
FF = 68%
FF = 40%
FF = 28%
FF = 51%
FF = 68%
FF = 46%
FF = 35%
FF = 23%
FF = 17%
FF = 14%
FF = 9.5%
FF = 7.0%
FF = 4.3%
FF = 3.3%
Fig. S16

## Slide 17
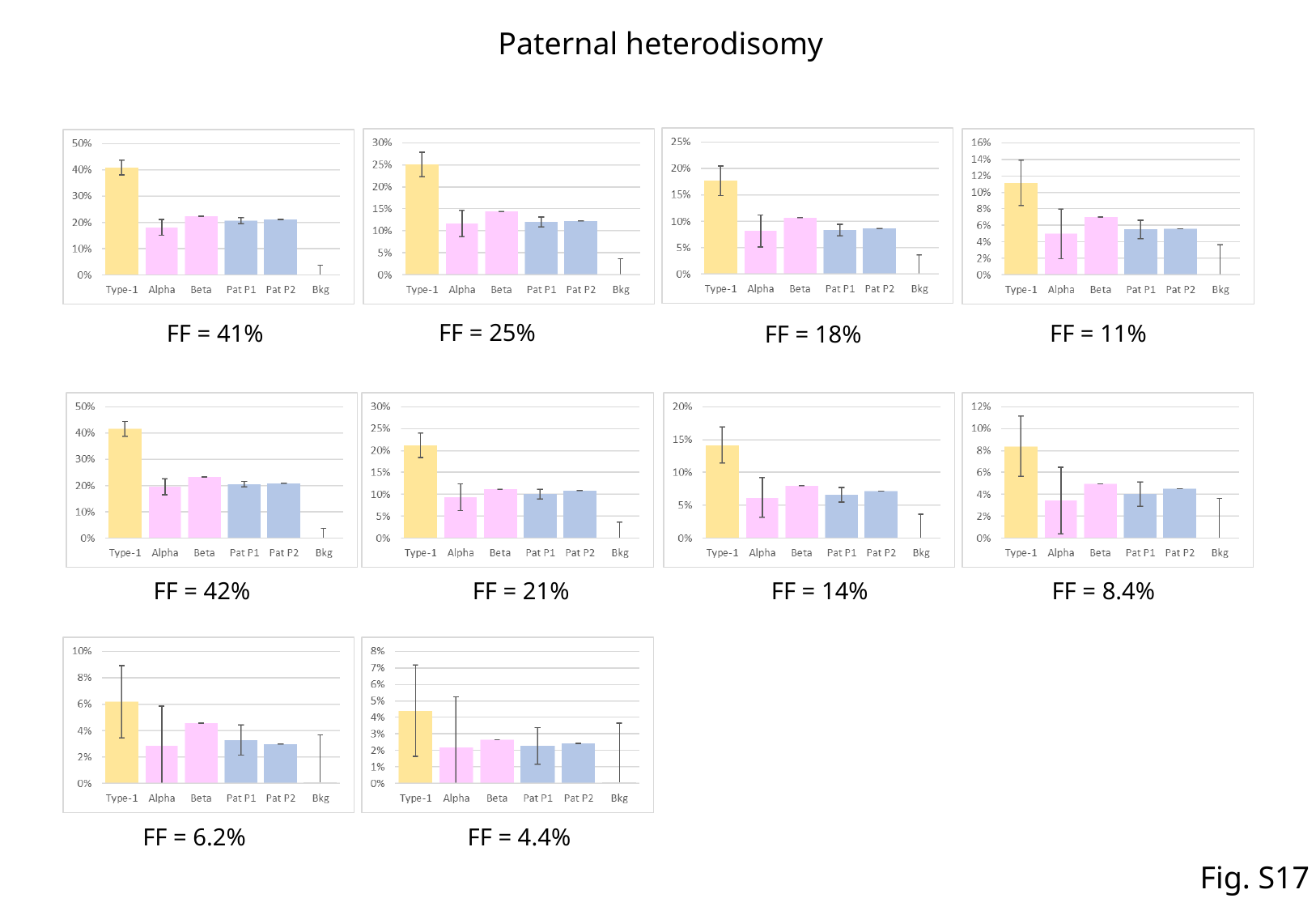

Paternal heterodisomy
FF = 25%
FF = 41%
FF = 11%
FF = 18%
FF = 42%
FF = 21%
FF = 14%
FF = 8.4%
FF = 6.2%
FF = 4.4%
Fig. S17

## Slide 18
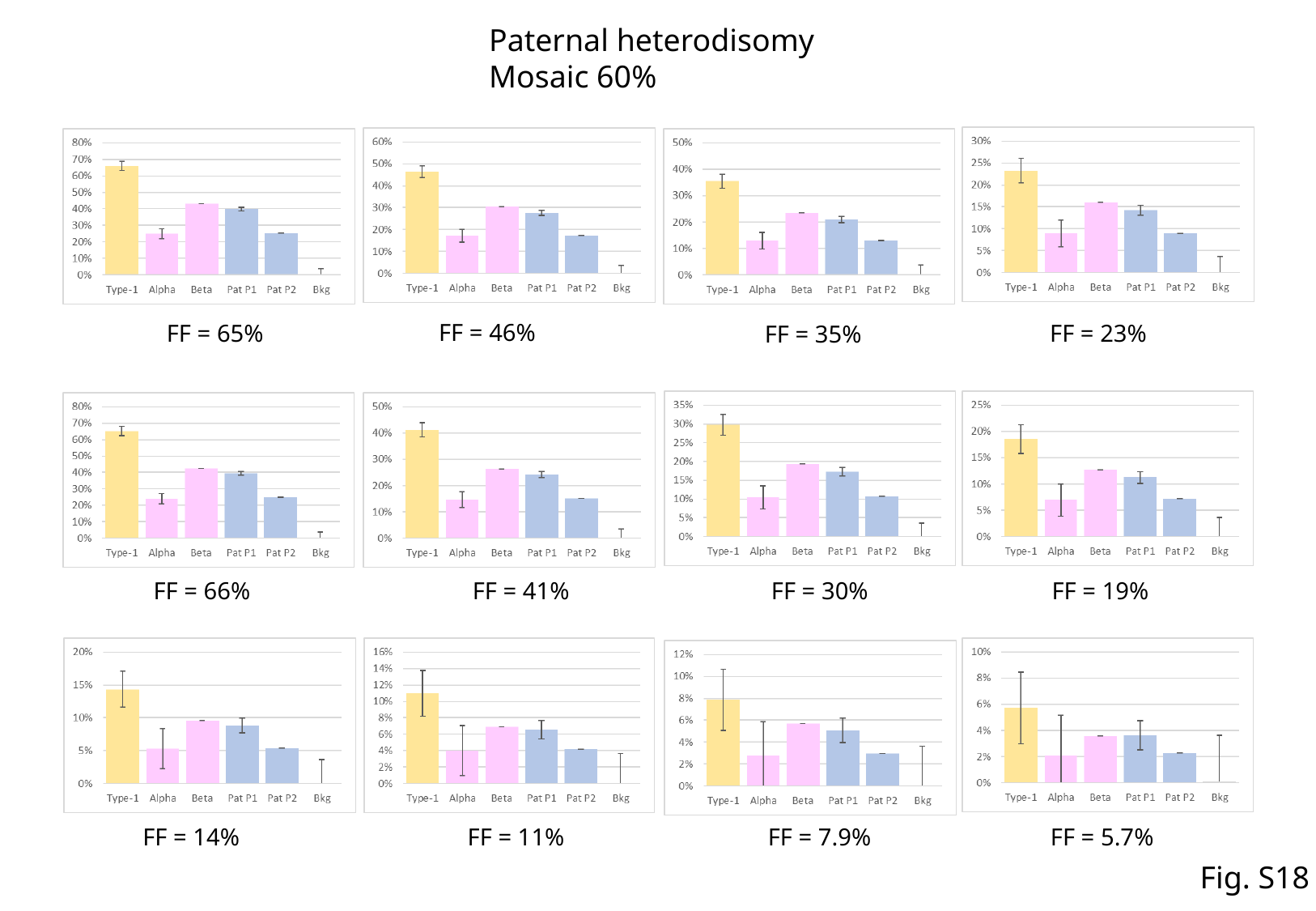

Paternal heterodisomy
Mosaic 60%
FF = 46%
FF = 65%
FF = 23%
FF = 35%
FF = 66%
FF = 41%
FF = 30%
FF = 19%
FF = 14%
FF = 11%
FF = 7.9%
FF = 5.7%
Fig. S18

## Slide 19
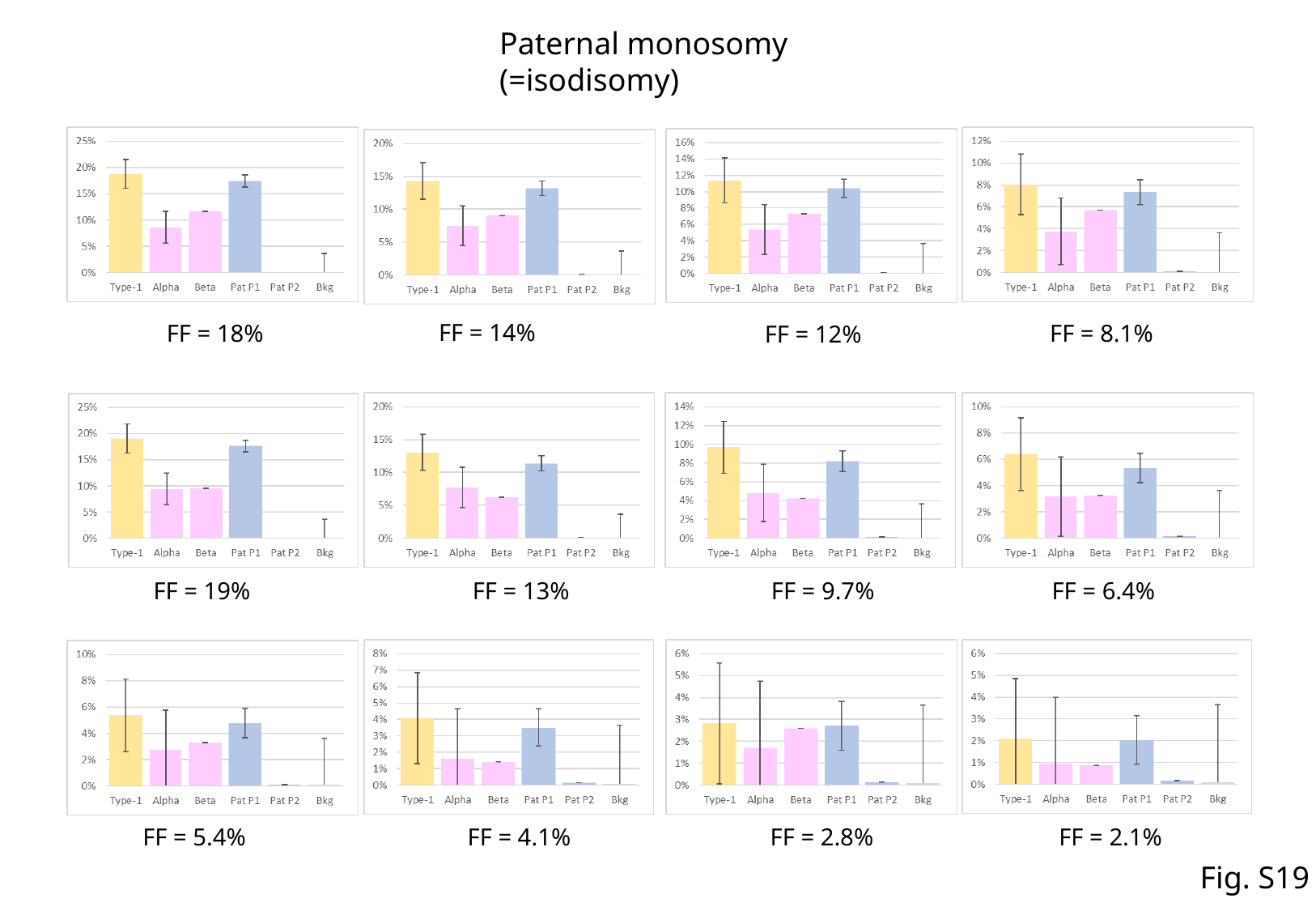

Paternal monosomy (=isodisomy)
FF = 14%
FF = 18%
FF = 8.1%
FF = 12%
FF = 19%
FF = 13%
FF = 9.7%
FF = 6.4%
FF = 5.4%
FF = 4.1%
FF = 2.8%
FF = 2.1%
Fig. S19

## Slide 20
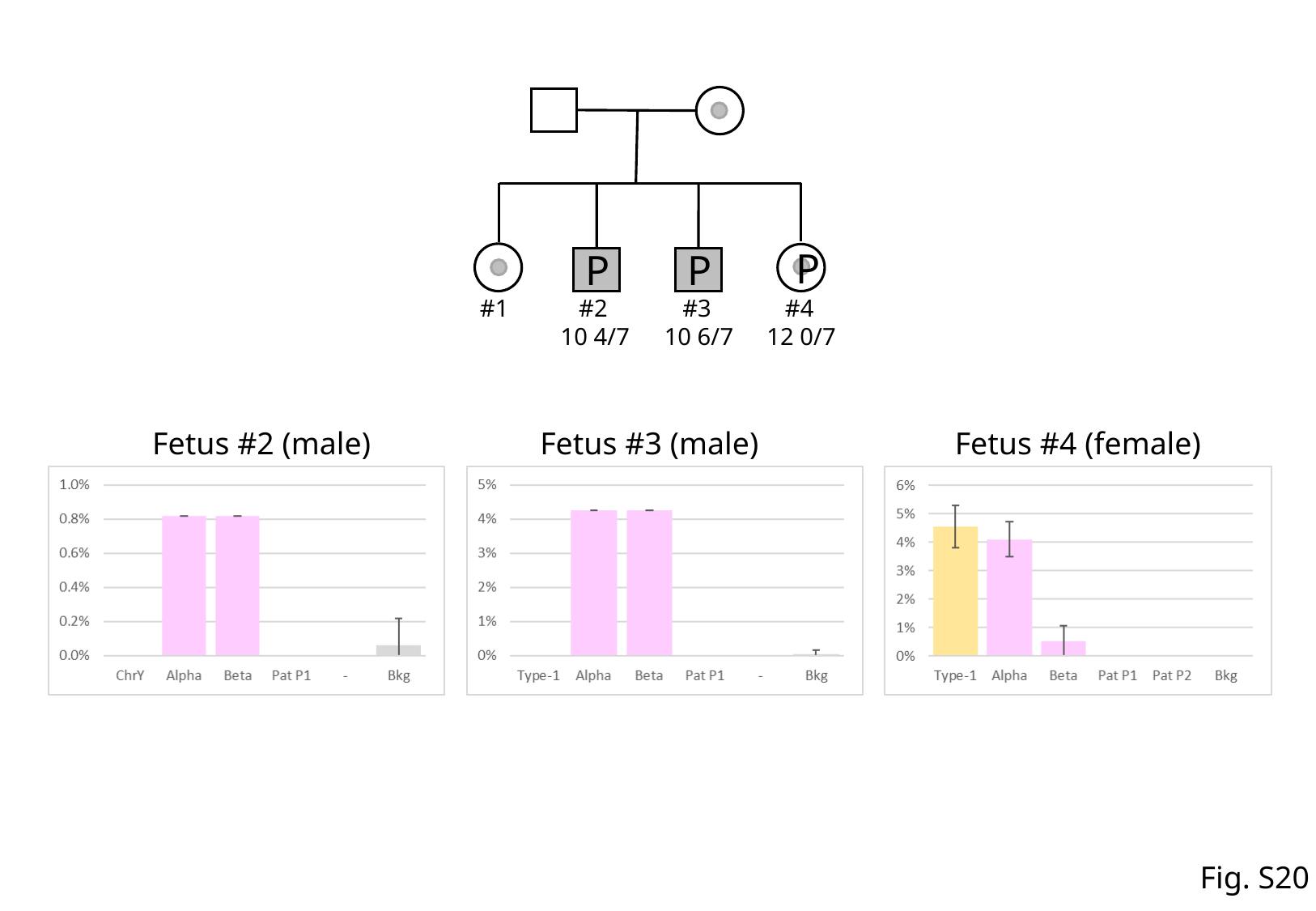

P
P
P
#1
 #2
10 4/7
 #3
10 6/7
 #4
12 0/7
Fetus #2 (male)
Fetus #3 (male)
Fetus #4 (female)
Fig. S20

## Slide 21
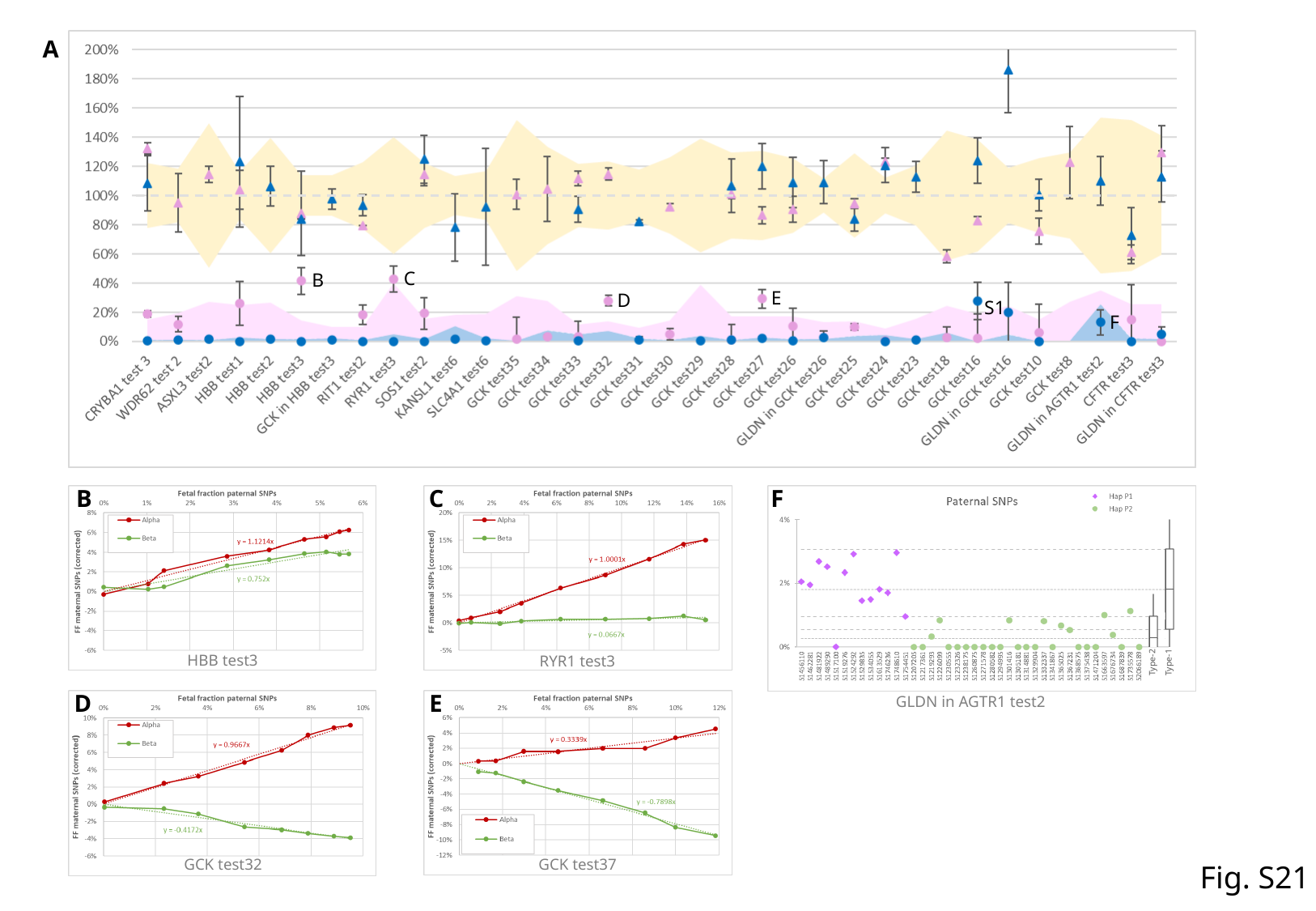

A
C
B
E
D
S1
F
F
Type-2
Type-1
GLDN in AGTR1 test2
B
C
HBB test3
RYR1 test3
D
E
GCK test37
GCK test32
Fig. S21
